# Astrocyte-specific knockout of YKL-40/*Chi3l1* reduces Aβ burden and restores memory functions in 5xFAD mice

**DOI:** 10.1186/s12974-023-02970-z

**Published:** 2023-12-02

**Authors:** Xiaoyan Zeng, Stanley K. K. Cheung, Mengqi Shi, Penelope M. Y. Or, Zhining Li, Julia Y. H. Liu, Wayne L. H. Ho, Tian Liu, Kun Lu, John A. Rudd, Yubing Wang, Andrew M. Chan

**Affiliations:** 1 School of Biomedical Sciences, The Chinese University of Hong Kong, Room G03, Lo Kwee-Seong Integrated Biomedical Sciences Building, Hong Kong SAR, China; 2 School of Life Science and Technology, Weifang Medical University, Shandong, China; 3 Department of Food Science and Nutrition, The Hong Kong Polytechnic University, Hong Kong SAR, China

**Keywords:** YKL-40, Astrocyte, Amyloid-beta, Alzheimer’s disease

## Abstract

Glial cell-mediated neuroinflammation and neuronal attrition are highly correlated with cognitive impairment in Alzheimer’s disease. YKL-40 is a secreted astrocytic glycoprotein that serves as a diagnostic biomarker of Alzheimer’s disease. High levels of YKL-40 are associated with either advanced Alzheimer’s disease or the normal aging process. However, the functional role of YKL-40 in Alzheimer’s disease development has not been firmly established. In a 5xFAD mouse model of Alzheimer’s disease, we observed increased YKL-40 expression in the cerebrospinal fluid of 7-month-old mice and was correlated with activated astrocytes. In primary astrocytes, Aβ_1-42_ upregulated YKL-40 in a dose-dependent manner and was correlated with PI3-K signaling pathway activation. Furthermore, primary neurons treated with YKL-40 and/or Aβ_1-42_ resulted in significant synaptic degeneration, reduced dendritic complexity, and impaired electrical parameters. More importantly, astrocyte-specific knockout of YKL-40 over a period of 7 days in symptomatic 5xFAD mice could effectively reduce amyloid plaque deposition in multiple brain regions. This was also associated with attenuated glial activation, reduced neuronal attrition, and restored memory function. These biological phenotypes could be explained by enhanced uptake of Aβ_1-42_ peptides, increased rate of Aβ_1-42_ degradation and acidification of lysosomal compartment in YKL-40 knockout astrocytes. Our results provide new insights into the role of YKL-40 in Alzheimer’s disease pathogenesis and demonstrate the potential of targeting this soluble biomarker to alleviate cognitive defects in symptomatic Alzheimer’s disease patients.

**Supplementary Information:**

The online version contains supplementary material available at 10.1186/s12974-023-02970-z.

## Introduction

Alzheimer’s disease (AD) is the most common form of dementia, characterized by progressive attrition of neuronal subsets resulting in impaired neuroplasticity and a continuum of clinical features ranging from mild cognitive impairment to complete cognitive and physical incapacitation [1, 2]. Amyloid plaques and tau-associated neurofibrillary tangles are two key diagnostic hallmarks of AD [3–5]. However, previous attempts to restore cognitive deficits in AD patients by targeting amyloid-beta (Aβ) have not been effective. The recently FDA-approved Aβ-directed monoclonal antibody drug Lecanemab is the latest in a long line of therapeutics that aimed to reduce the presumptive neurotoxic effects of Aβ [6].

Neuroinflammation plays a prominent role in AD progression [7–9]. In early AD, Aβ-producing neurons may initiate neuroinflammation by attracting microglia and stimulating the production of pro-inflammatory cytokines and chemokines that aggravate AD progression. In late AD, fully activated microglia surrounding amyloid plaques exert their phagocytic functions to clear toxic Aβ aggregates. Thus, neuroinflammatory processes can have detrimental or beneficial roles in AD disease progression.

YKL-40, a secreted chitin-binding lectin belonging to glycosyl hydrolase family 18, has been identified as a biomarker for advanced AD. High YKL-40 levels in cerebrospinal fluid (CSF) have been observed in early-stage AD patients [10], and are associated with acute and chronic neuroinflammation [11]. The central nervous system (CNS) YKL-40 appears to be derived from astrocytes and rare white matter neurons rather than infiltrating macrophages [10, 12]. YKL-40 transcription in astrocytes is stimulated through STAT3 and RelB/p50 transcriptional complexes by IL-1β and IL-6 released from macrophages [13]. However, the regulation and the biological function of YKL-40 in AD have not been firmly defined. Pathologically, elevated YKL-40 expression is associated with cortical thinning, hippocampal atrophy, and cognitive decline [14]. Mouse models of AD exhibit increased expression of chitinase-3 like 1 (*Chi3l1*), a mouse homologue of human YKL-40 [15]. *Chi3l1* was mainly expressed in astrocytes in the mouse brain, and an increased number of *Chi3l1*-positive reactive astrocytes was reported in advanced AD and associated with IBA1-positive microglia [16]. More recently, the characterization of a YKL-40-knockout mouse line revealed that YKL-40 suppressed glial cell-mediated phagocytosis and that YKL-40 expression is regulated by the circadian clock [17]. However, how YKL-40 regulates the Aβ processing and degradation are still unclear.

Toxic Aβ peptides are cleared in AD brains by multiple mechanisms. They could be taken up by phagocytosis or endocytosis through binding to lipoprotein receptor-related protein 1 (LRP-1) on astrocytes [18], triggering receptor expressed on myeloid cells 2 (TREM-2) on macrophages [19], or formyl peptide receptors (FPR) on microglia [20, 21]. Alternatively, Aβ peptides could be degraded by multiple proteases in the cytoplasm or extracellular space including neprilysin [22], cathepsin B [23], and α1-antichymotrypsin [24]. Autophagy, which has been implicated in the degradation of aggregated proteins, has also been shown to play a role in Aβ clearance in both microglia and astrocytes [25]. Finally, the ability of astrocytes to send out extensive processes to blood vessels allows the clearance of Aβ from the brain to the blood circulation [26]. The functional role of YKL-40 in Aβ processing and degradation has not been clearly defined. In this study, we report a significant enhancement in memory functions associated with astrocyte-specific knockout of YKL-40 and uncovered endocytic genes that may explain these biological effects.

## Materials and methods

### Mice

B6SJL-Tg(APPSwFlLon, PSEN1*M146L*L286V)6799Vas/Mmjax (stock#034840; 5xFAD hereafter) [27, 28], and B6;FVB-Tg(Aldhl11-cre/ERT2)1Khakh/J (stock#029655; Aldh hereafter) were purchased from the Jackson Laboratory (Bar Harbor, Maine). A YKL-40/*Chi3l1* conditional knockout mouse strain, B6-*Chil1*^*tm1amc*^/Cyagen was generated by a commercial source (Cyagen, Inc.) (*Chi3l1*^fl^ hereafter). A knockout cassette carrying a *Neo*^r^ gene was placed between exon 5 and 6 with flox sites flanking exon 5. Cre-mediated recombination event will eliminate exon 5 in the knockout allele. The above three mouse strains were maintained at the Chinese University of Hong Kong. Separately, a conventional YKL-40/*Chi3l1* mouse line, C57BL/6 J-Chil1em1cyagen (*Chi3l1*^−/−^ hereafter), was also purchased from Cyagen, Inc. and maintained in the animal center of Weifang Medical University. All mouse strains were backcrossed into a C57BL/6 J genetic background for at least 5 generations. To generate various YKL-40/*Chi3l1* knockout in 5xFAD experimental groups, double heterozygous Aldh;*Chi3l1*^+/fl^ and 5xFAD;*Chi3l1*^+/fl^ mice were crossed to derive all experimental groups.

### Primary cultures

For astrocytes, cortices of postnatal day 0 to 1 (P0-P1) pups were collected and triturated 3 times and resuspended with 2 ml of DMEM + 1% Pen/Strep + 5% fetal bovine serum (FBS, Gibco) and plated onto two 60-mm dishes coated with poly-D-lysine (PDL) (Sigma) and incubated at 37 °C, 5% CO_2_. For primary neurons, cortices or hippocampi from embryonic day 17.5 (E17.5) mice were triturated in ice-cold dissociating medium (DMEM + 1% Pen/Strep + 1% Antimycin/Antibiotic–Antimycotic, CAISSON Labs, ABL02-10) and 2 × 10^6^ cells were seeded onto PDL coated 6-well dish in neurobasal medium (Gibco) + 1% Pen/Strep + 1% Antimycin/Antibiotic–Antimycotic + 2% B-27 Supplement (Gibco) + 0.25% L-Glutamine (200 mM) (Gibco). One day later, all wells were treated with 1 mM AraC (Sigma Aldrich). Half of the medium was changed every 3–4 days (without AraC).

### Mouse dissection

Mice were anesthetized with ketamine/xylazine and head was fixed on a stereotaxic apparatus. Mouse skin was incised longitudinally to expose the dura over the cisterna magna. Then, the siphon glass tube was pushed into the cisterna magna and a total of 15–20 μl CSF was collected for each mouse. Next, the thoracic cavity was exposed, and blood was collected using a syringe through the right atrium and was immediately transferred into an EDTA treated 1.5 ml Eppendorf tube.

### Mouse YKL-40/Chi3l1 ELISA immunoassay

Mouse Chitinase 3-like 1 Quantikine ELISA kit (R & D, MC3L10) was used to determine YKL-40/Chi3l1 expression levels in CSF and plasma of 4-, 7-, 9-, and 16-month-old WT and 5xFAD mice according to the manufacturer’s instructions. A microplate reader was used to determine the OD 450-nm values within 30 min. All standards, control, and samples were assayed in duplicates. The relative CSF and plasma YKL-40/Chi3l1 expression were normalized with total protein measured by the BCA kit.

### Morris water maze

Male mice from 6.5-month- to 7.5-month-old were tested. Mice went through 1 day of cued training, 4 days of acquisition training, and a final day of probe trial. 5 trials were created on Day 0 to train mice to reach a visible platform. This was followed by 4 days of acquisition training with mice released at different release points to locate the submerged platform. Probe Trial was conducted on Day 5 to evaluate spatial memory ability. Only 1 trial that lasted for 60 secs was created for each mouse with no platform zone, and with the same release direction. EthoVision XT 11.5 software was used to track the number of entries, escape latency, swimming speed, and total path length. The tank was divided into 4 quadrants and was calibrated in the computer software to create physical distance information from pixel-based information.

### Western blotting analysis

Cells were solubilized in RIPA buffer and diluted with 5X Loading buffer plus 2-mercaptoethanol and boiled. Approximately 5–10 μg protein samples and PageRuler Pre-stained Protein Ladder (Thermo Fisher Scientific) were resolved on SDS-PAGE gel. Following transfer onto Nitrocellulose membrane (Bio-Rad), the membrane was incubated in blocking solution (5% milk) and probed with primary antibodies in 5% milk at 4 °C overnight. Bound antibodies were detected with peroxidase-conjugated secondary antibodies (in 5% milk) for at least 1 hour (h) at room temperature. The membranes were developed by Enhanced Chemiluminescence. The membranes were exposed to autoradiography film in a dark room or directly detected using ChemiDoc Touch Imaging System (Bio-Rad).

### Immunofluorescence staining

Each well of primary neurons in 12-well plates was fixed with 1 ml of 4% paraformaldehyde (PFA) (Sigma-Aldrich) at room temperature for 20 min. The neurons were permeabilized and blocked with 5% goat serum + 0.05% Triton X-100 in PBS for 60 min at room temperature. Primary antibodies were diluted to working concentration with 5% goat serum + 0.05% Triton X-100 in PBS and incubated at 4 °C, overnight. All neurons were probed with Alexa-conjugated secondary antibodies in 5% goat serum + 0.05% Triton X-100 in PBS for 1 h, washed and stained with DAPI (1 μg/mL) (Sigma-Aldrich) in PBS for 5–10 min at room temperature. Stained neurons were mounted with VECTASHIELD Mounting Medium (VECTOR) covered by coverslips and observed using Olympus SP8 Confocal System. Captured images were analyzed using ImageJ Fiji software. Primary astrocytes were cultured in 8-well chambers (3 × 10^4^ per well) for 24 h. Cells were fixed with 4% paraformaldehyde solution, blocked with 3% BSA and 0.2% Triton X-100 at room temperature for 1 h, and then incubated with primary antibody overnight at 4 °C. After washing, the cells were incubated with the secondary antibodies at room temperature for 1 h. The nuclei were then stained with DAPI and images were acquired by confocal microscopy.

### MTS/PMS cell viability assay

Primary neurons were seeded at 10^4^ cells/well in 96-well plates coated with 10 mg/mL PDL. Neurons were treated with different doses of Aβ and YKL-40 for 48 h and 72 h. 3-(4, 5-dimethylthiazol-2-yl)-5-(3-carboxymethoxyphenyl)-2-(4-sulfophenyl)-2H-tetrazolium (MTS) powder (Promega) was dissolved in PBS (2 mg/mL), pH6.0 ~ 6.5. PMS powder (Sigma) was also dissolved in PBS at a concentration of 0.92 mg/mL. The MTS / PMS solution was mixed at a ratio of 20 (MTS):1(PMS). Next, MTS/PMS solution was mixed with phenol red-free DMEM medium (Gibco) at the ratio of 1:5 and added to neurons. After incubating at 37 °C for 4 h, the OD values were measured at 490 nm on a spectrophotometer (Bio-Rad).

### Drugs treatment

Primary astrocytes were treated with 1 mM tamoxifen (Sigma-Aldrich) for three consecutive days. For in vivo experiments, mice were intraperitoneally injected with 100 µl tamoxifen (20 mg/mL in corn oil) for 7 consecutive days. Lyophilized Aβ_1-42_ peptide powder (AnaSpec) was dissolved in 80 μl 1% NH_4_OH, then immediately diluted with 1X PBS to a concentration of 1 mg/mL (221.5 mM). After vortexing, large aggregates were removed by centrifugation (14,000 × g at RT for 15 min), and preparations were stored at − 20 °C. 1% NH_4_OH/PBS solution was used to dilute the Aβ stock to a working concentration.

### Microelectrode array recoding (MEA)

Primary cortical neurons were harvested from E17.5 mice and seeded on microelectrode arrays (MEAs) (Multichannel System) for 24 days. The neuronal spontaneous electric activity was recorded beginning at DIV14 using an MEA2100 MultiChannel Systems on a heated stage at 37 °C. Neurons exhibited mature spikes (around DIV18) were exposed to 10 μM Aβ, 400 ng/mL YKL-40, and 10 μM Aβ + 400 ng/mL YKL-40, respectively. MEA recordings were measured at DIV14 (baseline), DIV19 (24 h after drug treatment), and DIV21 (72 h after drug treatment). The spikes were normalized to the pre-treatment activity of each array.

### Quantitative RNA sequencing and data analysis

Primary astrocytes isolated from wild-type mice and YKL-40 knockout mice were cultured in DMEM with 10% FBS and lysed in 1 ml TRIZOL™ Reagent (Invitrogen). Total RNA was extracted following the manufacturer’s instructions. Then, RNA samples were subjected to quantitative RNA sequencing by Genesky (Genesky Biotechnologies Inc., Shanghai). DEGs with threshold values of log2 fold-change (FC) > 1 and *p*-value less than 0.05 were captured and presented in the volcano map. For functional enrichment analysis, all DEGs were mapped to the Kyoto Encyclopedia of Genes and Genomes (KEGG) database, and significantly enriched KEGG pathways were searched. Furthermore, a gene set enrichment analysis (GSEA) (Broad Institute) tool kit was used to obtain the enrichment score for defined gene sets, such as GO gene sets and KEGG pathway gene sets.

### Aβ uptake and degradation by astrocytes

Primary astrocytes were seeded in 96-well culture plates (2 × 10^4^ per well) with 10% FBS-containing medium for 24 h. The cells were treated with 0.1 μM HiLyte™ Fluor 555-labeled Aβ42 (AnaSpec) for 24 h. The fluorescence intensity was determined at 540 nm excitation and 570 nm emission using a fluorescence microplate reader (Molecular Device, SpectraMax Gemini EM).

### Intracellular lysosomal pH detection

The intracellular pH of astrocytes was detected using the ratiometric probe LysoSensor™ Yellow/Blue DND-160 (Invitrogen, L7545). After treatment with 5 μM Aβ_1-42_ for 96 h, astrocytes were rinsed with PBS and dye was added according to the manufacturer's protocol, followed by intracellular pH measurement with a fluorescence microplate reader. Fluorescence intensity was detected at EX = 385 nm EM = 540 nm and EX = 330 nm EM = 440 nm.

### Flow cytometry

Primary astrocytes isolated from wild-type mice and YKL-40 knockout mice were prepared in a 6-well plate (1 × 10^5^ cells per well) 24 h before the assay. The cells were incubated with 0.1 μM Aβ42-555 or APC Beads (BD, 661620) for 24 h at 37 °C. After incubation, the cells were washed three times with cold PBS and then suspended in PBS. The cell suspension was gravity filtered through 35 μm nylon mesh prior and then introduced to a flow cytometer. The autofluorescence of the cells incubated without Aβ42-555 or APC Beads was used as a control.

### Data analysis

The mean surface area, integrated density, and synapse density of confocal images were measured by ImageJ Fiji software**.** GraphPad Prism software (Version 9.0, San Diego, CA, USA) was used to analyze the statistical significance and was used to construct various graphs. Neuronal spikes were identified and analyzed using Signal Processing Toolbox in MATLAB (2020a; MathWorks, California, United States)(see codes for spike analysis in Supplemental Materials section).

For MEA data analysis, recordings were performed for 5 min using threshold crossing methods at a 250000 Hz sampling rate. The raw data were filtered at 300 Hz high pass cut-off frequency using MC_Rack software. Thresholds were set at 3 times the standard deviation noise level of the individual channels. The pre-spike and post-spike durations were both set to 2 ms. Only channels that showed 10 or more spikes and low baseline noise (< 40 V) were included for analysis. We called electrodes that met these criteria “active electrodes”. All active channels of each drug and time point were pooled together for their respective statistical analyses. Significant differences between drugs at a given time point were tested by analysis of variance (ANOVA) followed by Dunnett’s multiple comparison post hoc test with a significance threshold of α = 0.05. Data were presented as mean ± SEM, n = 100–120 neurons from three independent experiments.

For Sholl Analysis, the image stacks of GFAP and MAP2 staining were reconstructed as binarized representations, and the Sholl method of concentric circles was used to determine astrocyte/neuron ramification [29]. Each cell was manually tracked to exclude adjacent cells by Simple Neurite Tracing (SNT) and analyzed by selecting the center of its soma and then running the *Sholl* analysis procedure, which counts the number of intersections at circles of increasing radii from the center. The maximal ring with an intersecting process (max distance), the mean number of intersections (branching complexity) for all circles, and the ramification index were calculated for each cell. Astrocytes: start radius (μm): 0.552; step size (μm): 1.664; samples per radius: 2. Neurons: start radius (μm): 3; step size (μm): 3; samples per radius: 2. Data were shown as mean ± SEM of 70–75 astrocytes from five independent experiments and 50–70 neurons from four independent experiments. GraphPad Prism software (Version 9.0, San Diego, CA, USA) was used to analyze statistical significance and graph constructions.

## Statistical analysis

Quantitative data were represented as mean ± SEM. Differences between groups were tested with one-way ANOVA with Tukey’s post hoc comparisons, two-way ANOVA followed by Tukey’s multiple comparisons, and an unpaired t-test. Mann–Whitney test and Kruskal–Wallis test were used for non-parametric data when appropriate. Significance levels were defined as follows: *p < 0.05, **p < 0.01, ***p < 0.001.

## Results

### Increased astrocytic YKL-40 expression in 5xFAD mice

**Fig. 1:**
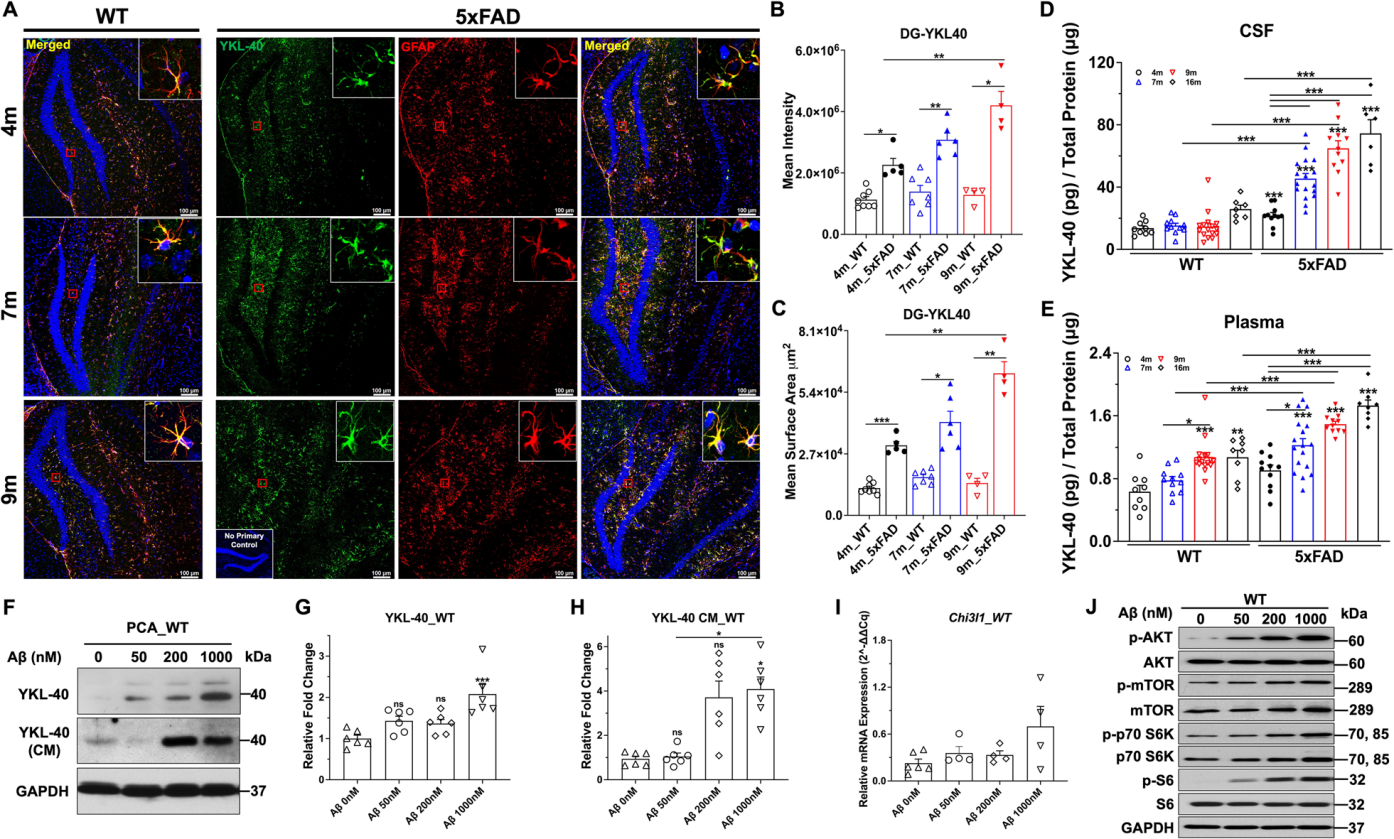
Elevated Astrocytic YKL-40 expression in 5xFAD mice. **A** Representative confocal images (Mag. 10X) of dentate gyrus region from 4-month (m), 7 m, and 9 m 5xFAD mouse brains immuno-stained with DAPI (blue signal), anti-YKL-40 (green signal), and anti-GFAP (red signal) antibodies. Scale bar, 100 μm. Mean intensity (**B**) and mean surface area (**C**) of YKL-40 signal in DG region were quantified from 4 m-, 7 m-, and 9 m- WT and 5xFAD mice. 4 m (n = 6), 7 m (n = 6), 9 m (n = 4). Data are mean ± SEM. *One-way ANOVA* with *Tukey’s *post hoc comparisons. *p < 0.05, **p < 0.01, ***p < 0.001. **D** YKL-40 expression level in cerebral spinal fluid (CSF) extracted from WT and 5xFAD mice. **E** YKL-40 expression level in plasma extracted from WT and 5xFAD mice. YKL-40 protein was measured by ELISA and data was normalized by total protein. Data are mean ± SEM of 6 to 17 animals. *One-way ANOVA* with *Tukey’s *post hoc comparisons. *p < 0.05, **p < 0.01, ***p < 0.001 compared to WT-4 m. **F** Western blotting analysis of YKL-40 expression level from astrocyte lysates and conditioned medium (CM) treated with 0, 50, 200, and 1000 nM Aβ_1-42_ for 72 h. GAPDH was used as an internal control. The relative YKL-40 expression level in CM was normalized with total protein. **G-H** Quantification of relative fold change of YKL-40. n = 6. Data are mean ± SEM. *One-way ANOVA* with *Tukey’s *post hoc comparisons. *p < 0.05, ***p < 0.001 compared to control. **I.** Relative *Chi3l1* gene expression from primary astrocytes treated with 0, 50, 200, and 1000 nM Aβ_1-42_ for 72 h. n = 3 to 6 from independent experiments. Data are mean ± SEM. **J.** Western blotting analysis of PI3-K pathway expression from astrocytes lysates treated with 0, 50 nM, 200 nM, 1000 nM Aβ_1-42_ for 72 h. GAPDH was used as internal control

5xFAD mouse strain was selected to unravel the role of YKL-40 in AD. The 5xFAD mouse strain expresses human *APP* and *PSEN1* transgenes with a total of five AD-linked mutations [30–32]. Immunostaining revealed extensive Aβ plaques in 5xFAD mice, even in the presymptomatic phase at 4 months old (Additional file 1: Fig. S1A). As expected, the plaque numbers in the dentatae gyrus (DG) region were significantly increased in 7- and 9-month-old 5xFAD mice (Additional file 1: Fig. S1B-C). Co-staining of DG and CA1 regions revealed extensive overlapping signals of GFAP and YKL-40 of 5xFAD mice (Fig. 1A). The YKL-40 levels showed a progressive increase with age from 4 to 9 months old (Fig. 1B, C). Furthermore, GFAP-positive reactive glial cells were detected in close proximity to Aβ-positive senile plaques (Additional file 1: Fig. S1B). Similar observations were detected in cortex, CA3, and thalamus (not shown). Further co-staining also revealed YKL-40-positive cells in the immediate vicinity of Aβ deposits (Additional file 1: Fig. S1D). These results imply an association between YKL-40 expression in astrocytes and amyloid plaque deposition. Because YKL-40 is a prognostic biomarker for AD, CSF and plasma were collected from 4-, 7-, 9-, and 16-month-old wild-type and 5xFAD mice. Using ELISA, we observed a significant (~ threefold) increase in soluble YKL-40 in the CSF of 5xFAD mice from 7 months onward (Fig. 1D). In WT mice, this increasing trend was not statistically significant. While similar increases in soluble YKL-40 were also detected in 5xFAD mice plasma, the ~ 1.5-fold increase was relatively modest compared with that observed in CSF (Fig. 1E). Next, primary astrocytes were treated with Aβ_1-42_ to mimic the in vivo condition of 5xFAD. Aβ_1-42_ induced a dose-dependent upregulation of intracellular and secreted YKL-40 in the conditioned medium (CM) of WT astrocytes at protein (Fig. 1F–H) and mRNA (Fig. 1I) levels.

Alterations in the PI3-K pathway have been shown to impair neurotransmission and long-term plasticity [33], and we have previously demonstrated a transcriptional link between PTEN and YKL-40 [34]. Primary astrocytes treated with Aβ_1-42_ induced activations of multiple components of the PI3-K pathway. In particular, both AKT and S6 were robustly activated by ~ 30-fold while both mTOR and p70 S6K were modestly activated by 2- to threefold (Fig. 1J and Additional file 1: Fig. S1E). In contrast, two members of the pro-inflammatory signaling pathway, JAK2 and STAT3, were not significantly activated (data not shown). Thus, the ability of Aβ_1-42_ to increase YKL-40 expression appears to be correlated with PI3-K activation state in primary mouse astrocytes.

### YKL-40 induces neurotoxicity in primary neurons

**Fig. 2:**
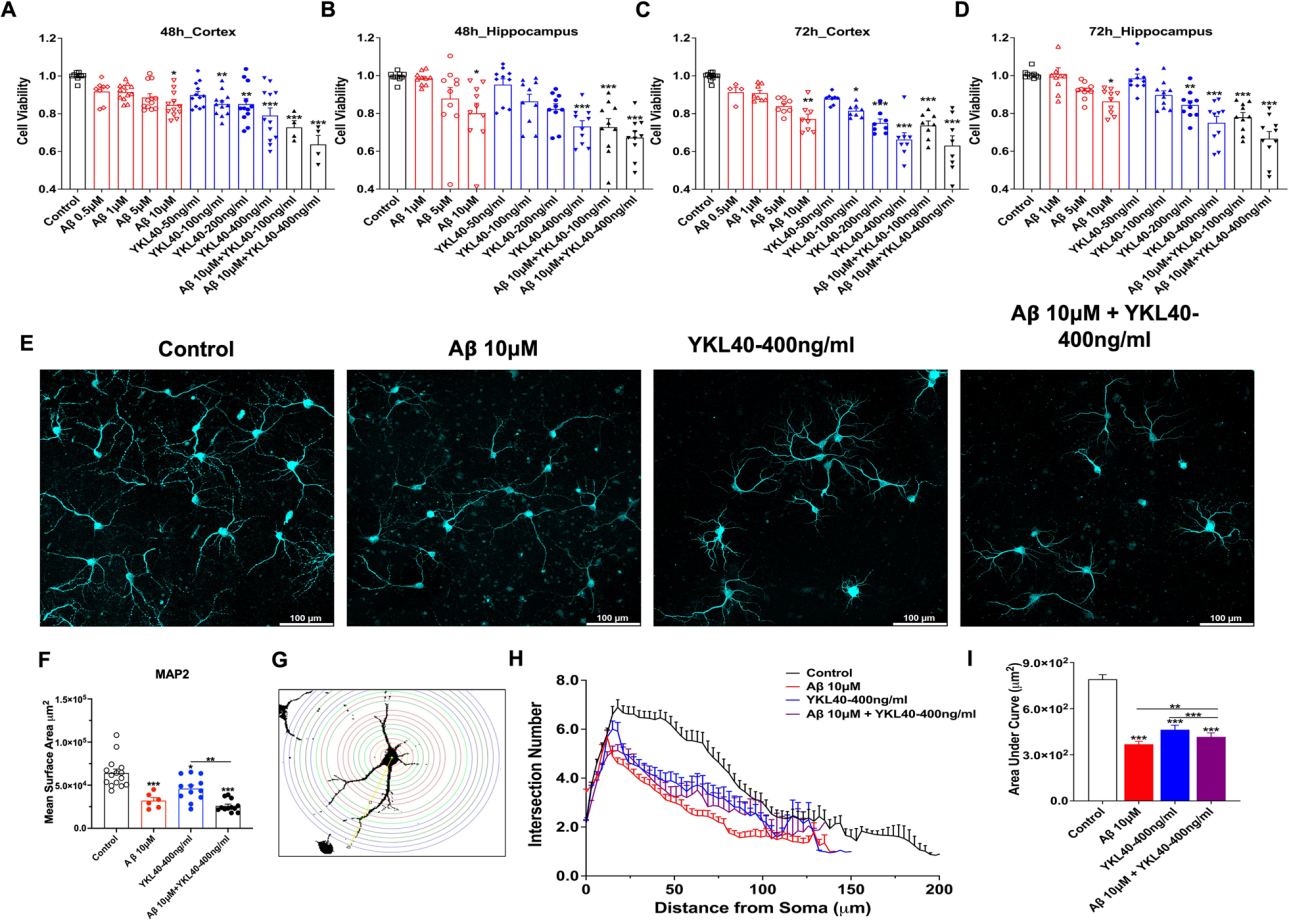
YKL-40 induces neuronal toxicity and dendritic degradation. Primary neurons were established from E17.5 wild type mice. **A–D** MTS assay was used to determine cell viability of DIV14 primary cultured cortex (Cor) & hippocampus (Hip) neurons treated with Aβ_1-42_ and/or YKL-40 for 48 or 72 h. Data are mean ± SEM. n = 4 to 12. *One-way ANOVA* with *Tukey’s *post hoc comparisons. *p < 0.05, **p < 0.01, ***p < 0.001 compared to control. **E** Confocal images (Mag. 10X) of primary neurons immuno-stained with an anti-MAP2 (cyan signal) antibody. Scale bars, 100 μm. **F** MAP2 mean surface area was quantified. Data are mean ± SEM. *One-way ANOVA* with *Tukey’s *post hoc comparisons. *p < 0.05, **p < 0.01, ***p < 0.001 compared to control. **G–I** *Sholl* Analysis (Image J) of dendritic intersection number from tracing images. Data are mean ± SEM of 50 to 70 neurons. *Two-way ANOVA* with *Tukey’s *post hoc comparisons. **p < 0.01, ***p < 0.001 compared to control

To examine whether YKL-40 modulates the neurotoxic effects of Aβ_1-42_ in neuronal cells, primary cortical and hippocampal neurons were cultured from E17.5 embryos. Treatments with increasing amounts of either Aβ_1-42_ (1–10 μM) or YKL-40 (100–400 ng/mL) resulted in significant dose- and time-dependent loss of cell viability by as much as 30% (Fig. 2A-D and Additional file 1: Fig. S2A). Combined treatments with two dose combinations (10 μM Aβ_1-42_ + 100 ng/mL YKL-40 or 10 μM Aβ_1-42_ + 400 ng/mL YKL-40) resulted in further decreases in cell viability by 40% (Fig. 2A–D and Additional file 1: Fig. S2A).

To better visualize the neuronal morphologies, treated cells were immuno-stained with anti-MAP2 antibody (Fig. 2E). Aβ_1-42_ or YKL-40, added either alone or in combination, caused clear neurite retraction and decreased branching, as reflected in the reductions in mean intensity and mean surface area by 50 to 60% (Fig. 2F). Individual neurons were traced using the Simple Neurite Tracing plugin, and the dendritic complexity was analyzed using Sholl analysis. As shown in Fig. 2G–I and Additional file 1: Fig. S2B–H, Aβ_1-42_ and YKL-40 added alone or in combination significantly decreased neuronal complexity, reducing the intersection number, soma perimeter, dendritic length, axon length, axon node numbers, dendrite node, and dendrite end. However, no synergistic effect between the two compounds was observed.

### Disturbed spontaneous electric activity and synaptic degradation in Aβ_1-42_- and YKL-40-treated primary neurons

**Fig. 3:**
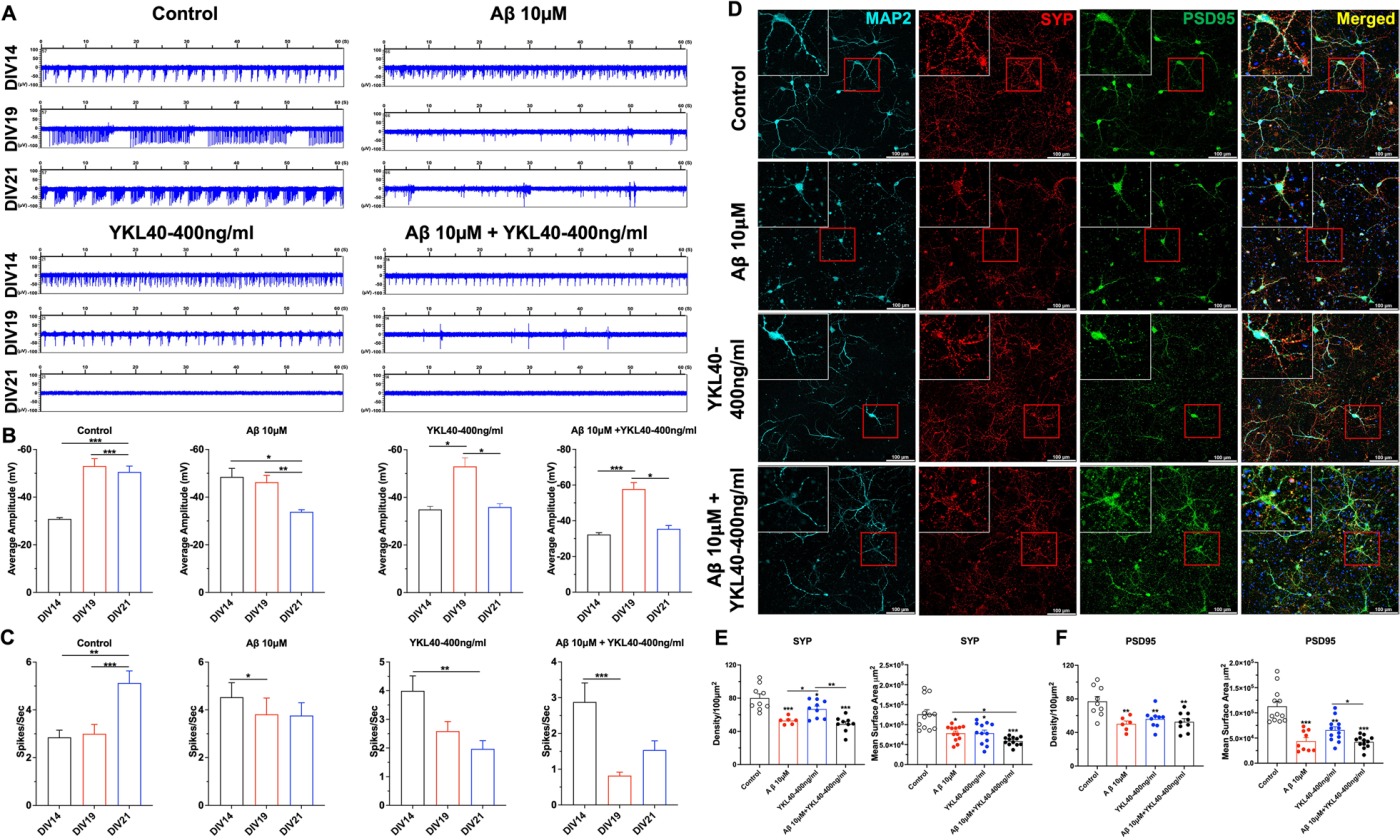
Impaired neuronal electric activities of Aβ_1-42_ and/or YKL-40 treated primary neurons. **A** Representative raw spike traces from four active electrode versus time after exposure to 10 μM Aβ, 400 ng/mL YKL-40, and 10 μM Aβ + 400 ng/mL YKL-40 in DIV18 cultures. **B–C** Quantified data showing time-dependent mean amplitude and spike rate for MEA neuronal cultures. Data are mean ± SEM of 100–120 neurons from three independent experiments. *One-way ANOVA* with *Tukey’s *post hoc comparisons. *p < 0.05, **p < 0.01, ***p < 0.001. **D** Confocal images (Mag. 10X) of primary neurons immuno-stained with anti-MAP2 (cyan signal), anti-PSD95 (green signal), and anti-SYP (red signal) antibodies. Scale bars, 100 μm. **E–F** Mean intensity and surface area were quantified. Mean surface area (n = 12), Density (n = 8) with at least 100 neurons included. Data are mean ± SEM. *One-way ANOVA* with *Tukey’s *post hoc comparisons. *p < 0.05, **p < 0.01

To explore whether Aβ_1-42_ and YKL-40 could alter the spontaneous firing activity of cortical neurons, neuronal activities were recorded using a 60-channel microelectrode array (MEA). As shown in Fig. 3A, four electrodes from each group were used to record the electrical activities of DIV14 to DIV21 neurons. As expected, the neuronal spontaneous spike rate in the control group progressively increased from DIV14 to DIV21 (Fig. 3A). However, when Aβ_1-42_ and YKL-40 were added alone or in combination, this spontaneous electrical parameter was significantly suppressed in DIV19 and DIV21 (Fig. 3A). In terms of spike amplitude, the spontaneous firing activity plateaued around DIV19 in the control group (Fig. 3B). Treatment with Aβ_1-42_ and/or YKL-40 caused suppression to baseline levels in DIV21 (Fig. 3B). For spike numbers per second, while a significant increase was observed in DIV21 in the control group, this enhancement was not detected in groups treated with Aβ_1-42_ or YKL-40 either alone or in combination. In fact, there were significant decreases in DIV19 neurons following treatment with Aβ_1-42_ or with a combination of Aβ_1-42_ + YKL-40; as well as DIV21 neurons following treatment with YKL-40 alone. (Fig. 3C).

The adverse effects exerted by YKL-40 or Aβ_1-42_ on the electrical properties of neurons could be caused by biochemical or structural defects in synapses. We explored whether the addition of YKL-40 influenced synaptic morphology. Synaptic density was measured using anti-SYP and anti-PSD-95 co-staining of primary cortical neurons at DIV14 (Fig. 3D). As shown in Fig. 3E, F, significant reductions in synaptic density by 1.2- to 1.6-fold were observed in neurons treated with either 400 ng/mL YKL-40 or 10 μM Aβ_1-42_ alone. However, no further decline was observed when YKL-40 was added simultaneously with Aβ_1-42_, suggesting that Aβ_1-42_ treatment and YKL-40 expression may share a common mechanism for inducing neurodegeneration.

### YKL-40 knockout reduces Aβ plaques and neuronal loss in 5xFAD mice

**Fig. 4:**
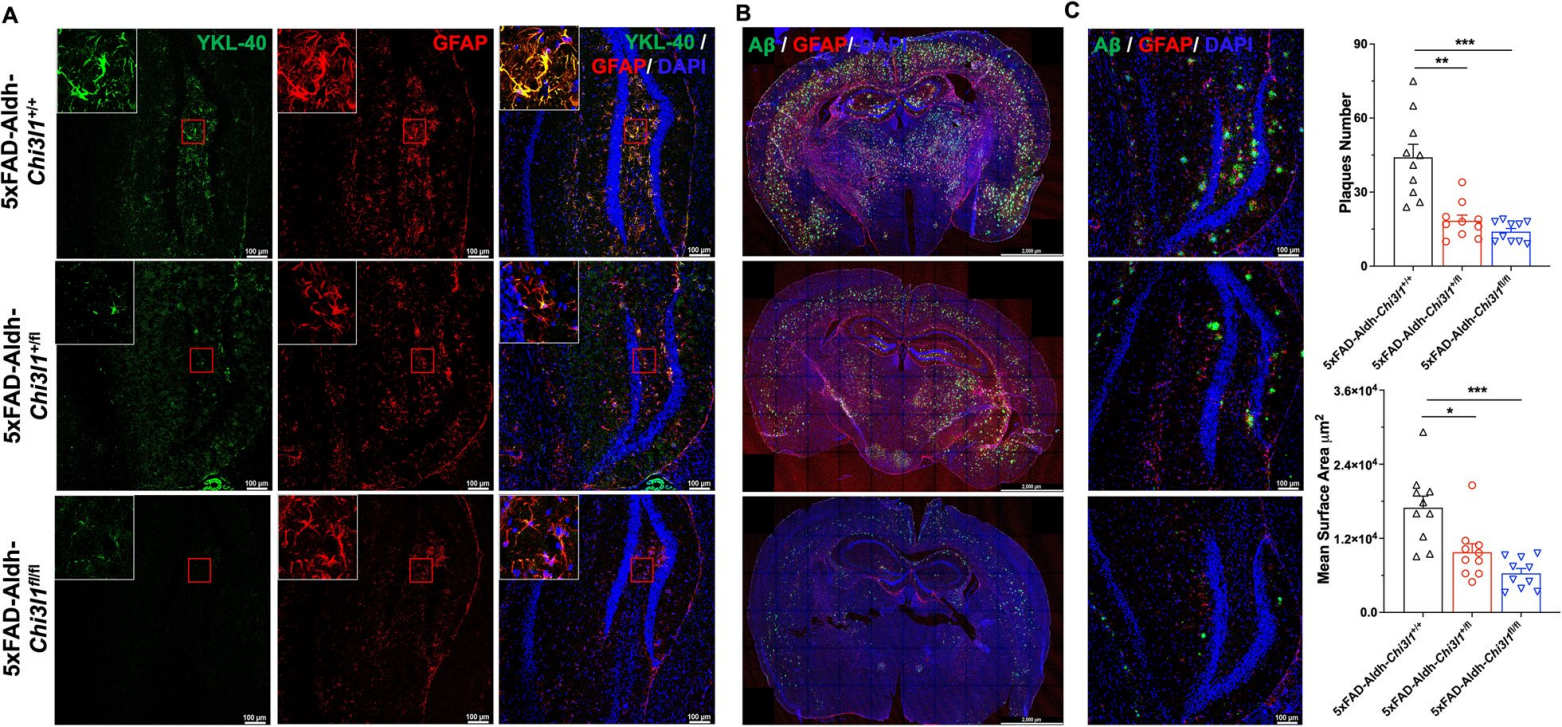
Knockout of YKL-40 in astrocytes reduces Aβ deposition in 5xFAD mice brain. All mice were IP injected with 20 mg/mL tamoxifen in corn oil (100 µl per mice/per day) for 7 consecutive days before experiments. Experiments were carried out 7 days after last tamoxifen injection. **A** Confocal images (Mag. 10X) of dentate gyrus region of 5xFAD;Aldh;*Chi3l1*^+/+^; 5xFAD;Aldh;*Chi3l1*^+/fl^; 5xFAD;Aldh;*Chi3l1*^fl/fl^ mouse brain immuno-stained with DAPI (blue signal), anti-YKL-40 (green signal), and anti-GFAP (red signal) antibodies. Scale bars, 100 μm. **B** Parallel brain sections were co-stained with DAPI (blue signal), anti-Aβ (green signal), and anti-GFAP (red signal) antibodies. **C** High magnification image of Dentate Gyrus regions. Quantification of plaque number from Aβ signals and mean surface area. Data are mean ± SEM. *One-way ANOVA* with *Tukey’s *post hoc comparisons. *p < 0.05, **p < 0.01, ***p < 0.001 compared to 5xFAD;Aldh;*Chi3l1*^+/+^

To explore the role of YKL-40 in 5xFAD, the *Chi3l1* (*YKL-40*) gene was conditionally deleted in astrocytes of 5xFAD mice. A transgenic mouse line, Aldh, that allows pan-astrocyte inducible knockout of genes was used (Additional file 1: Fig. S3A)[35]. The exon 5 of *Chi3l1* was deleted by tamoxifen injection one week prior to the experiment (Additional file 1: Fig. S3B, E). WT (Aldh;*Chi3l1*^+/+^) and heterozygous (Aldh;*Chi3l1*^+/fl^) mice had detectable YKL-40 expression in GFAP^+^ cells in both DG and TH (thalamus) regions (Additional file 1: Fig. S3F-G). In contrast, YKL-40 knockout mice (Aldh;*Chi3l1*^fl/fl^) had drastically reduced YKL-40 expression in GFAP^+^ cells, suggesting effective knockout of this gene (Additional file 1: Fig. S3F–G). Next, we crossed Aldh;*Chi3l1*^+/fl^ mice with 5xFAD;*Chi3l1*^+/fl^ mice to yield six experimental groups: 5xFAD;Aldh;*Chi3l1*^+/+^, 5xFAD;Aldh;*Chi3l1*^+/fl^, 5xFAD;Aldh;*Chi3l1*^fl/fl^, 5xFAD, WT;Aldh;*Chi3l1*^fl/fl^, and WT (Additional file 1: Fig. S3C). As expected, immunofluorescence analysis revealed the loss of YKL-40 expression in GFAP^+^ cells of 5xFAD;Aldh;*Chi3l1*^+/fl^ (heterozygous knockout) and 5xFAD;Aldh;*Chi3l1*^fl/fl^ (homozygous knockout) DG regions (Fig. 4A).

To determine the effect of YKL-40 knockout on Aβ_1-42_ accumulation in 5xFAD mice, immunofluorescence analysis was performed on brain tissues from 7-month-old WT and YKL-40-deficient mice. Significant global reductions in Aβ plaque were observed in both 5xFAD;Aldh;*Chi3l1*^+/fl^ 5xFAD;Aldh;*Chi3l1*^fl/fl^ mice (Fig. 4B). The plaque numbers and mean surface area were reduced by 66% and 58%, respectively in the DG regions of 5xFAD;Aldh;*Chi3l1*^fl/fl^ mice (Fig. 4C). Similar observations were detected in motor cortex + retrosplenial cortex (MOC), cortex (CTX), Cornu Ammonis 3 (CA3), and thalamus (TH) regions (Additional file 1: Fig. S4A, B).

**Fig. 5:**
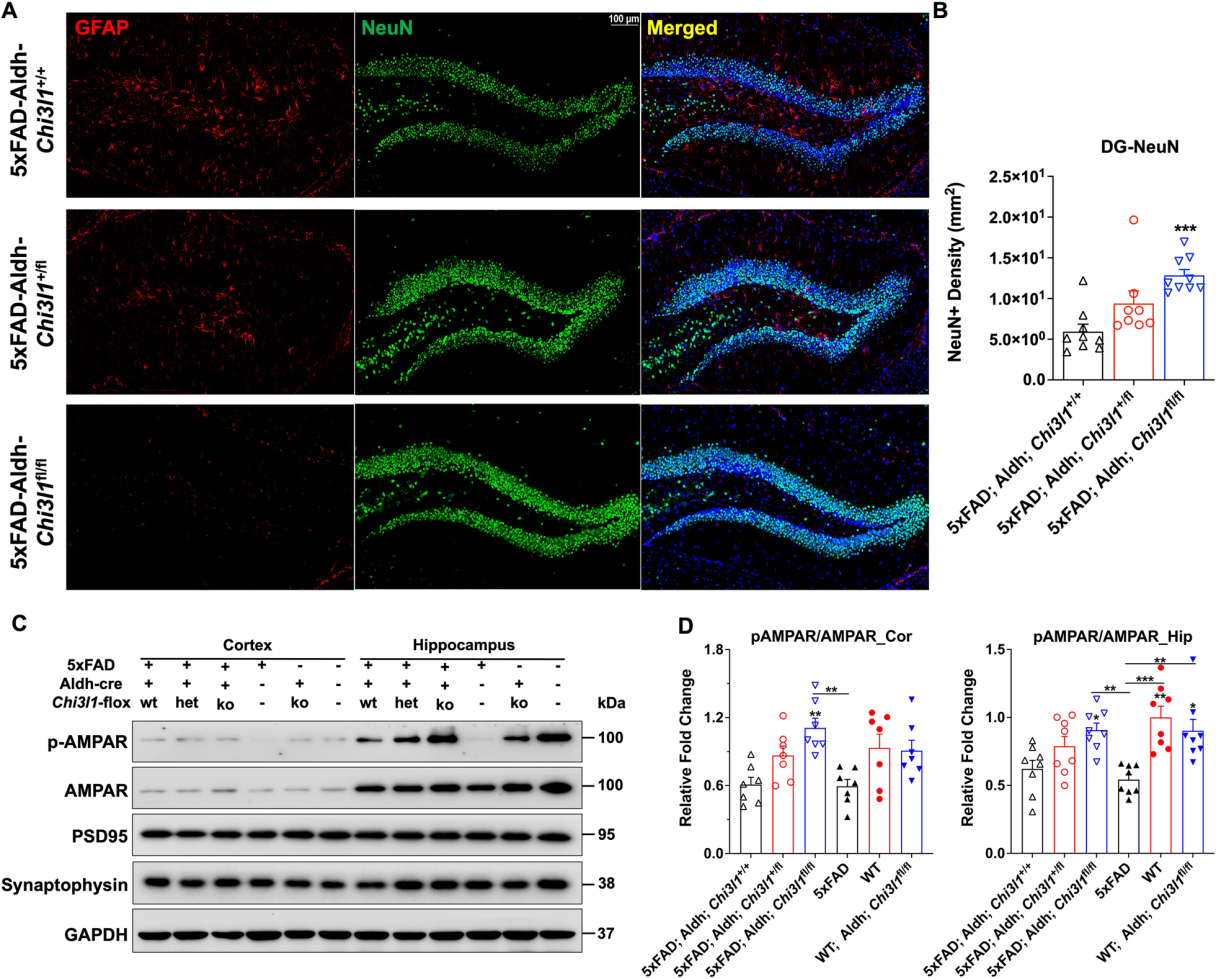
Depletion of YKL-40 in astrocytes prevents neuronal loss in the brain of 5xFAD mice. All mice were IP injected with 20 mg/mL tamoxifen in corn oil (100 µl per mice/per day) for 7 consecutive days before experiments. Experiments were conducted 7 days after last tamoxifen injection. **A** Confocal images (Mag. 10X) of dentate gyrus region 7-m mice brain immuno-stained with DAPI (blue signal), anti-NeuN (green signal), and anti-GFAP (red signal) antibodies. Scale bars, 100 μm. **B** Mean NeuN + density were quantified. Data are mean ± SEM of 7–9 brains (2–3 slices for each brain), then analysed by *One-way ANOVA* with *Tukey’s *post hoc comparisons. ***p < 0.001, compared to 5xFAD mice. DG: dentate gyrus. **C** Western blotting analysis of neuronal proteins in lysates isolated from cortex (Cor) and hippocampus (Hip). GAPDH was used as an internal control. **D** Western results were quantified for cortical (Cor) and hippocampal (Hip) samples. Data are mean ± SEM. n = 7. One-way ANOVA with Tukey’s post hoc comparisons. *p < 0.05, **p < 0.01, ***p < 0.001 compared to WT mice

NeuN is a marker of postmitotic neurons and loss of NeuN-positive cells is observed in mouse models of AD [36]. Immunostaining showed significant increased in NeuN immunoreactivity in 5xFAD;Aldh;*Chi3l1*^fl/fl^ knockout mice to a level similar to the WT (Fig. 5A, B). In addition, significant increases in p-AMAPR in both cortical and hippocampal regions were observed in YKL-40 knockout 5xFAD mice compared with control 5xFAD mice (Fig. 5C, D). As a major excitatory neurotransmitter receptor in CNS, disturbed AMPAR surface diffusion is thought to be a critical mechanism underlying glutamatergic synaptic plasticity [37]. However, YKL-40-depletion in 5xFAD mice failed to alter the expression of several synaptic markers, such as PSD95 and synaptophysin (SYP) (Fig. 5C). In fact, YKL-40 knockout only mildly increased SYP expression in the hippocampal lysates of 5xFAD mice (Fig. 5C).

Furthermore, we marked microglia and astrocytes with IBA1 and GFAP, respectively, and significant decreases in glial activation in multiple brain regions of YKL-40 knockout 5xFAD mice were observed via confocal microscopy (Additional file 1: Fig. S5A–D). Western blotting analysis of hippocampal and cortical lysates revealed elevated IBA1 and GFAP expression in 5xFAD mice compared with WT mice (Additional file 1: Fig. S5E–H). However, a significant decrease in IBA1 expression was only observed in the cortex of YKL-40 knockout 5xFAD mice (Additional file 1: Fig. S5G). This discrepancy may be due to the heterogeneity of cell types in bulk tissue sample analyses.

### YKL-40 astrocyte-conditional knockout rescues cognitive deficits in 5xFAD mice

**Fig. 6:**
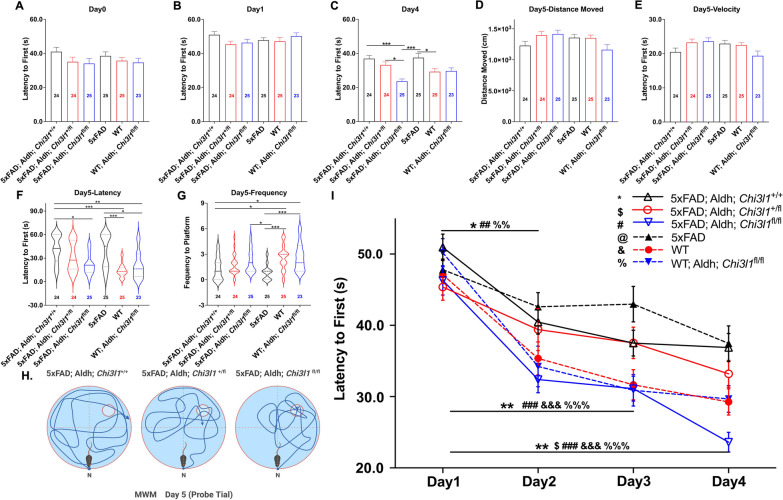
Knockout of YKL-40 in astrocytes attenuates cognitive deficits of 5xFAD mice. All mice were IP injected with 20 mg/mL tamoxifen in corn oil (100 µl per mice/per day) for 7 consecutive days before experiments. Experiments were carried out 7 days after last tamoxifen injection. Spatial memory and learning ability were tested using Morris Water Maze. **A-C** Quantified escape latency at day 0 (visible platform), day 1, and day 4. **D–G** Quantified total length, mean speed, escape latency, and number of entries at day 5 (probe trial). Data are mean ± SEM. *One-way ANOVA* with *Tukey’s *post hoc comparisons. *p < 0.05, **p < 0.01, ***p < 0.001. **H** Schematic representation of Morris water maze. **I** Quantified escape latency from day 1 to day 4 (training trials). Data are mean ± SEM. n = 23 ~ 25. *One-way ANOVA* with *Tukey’s *post hoc comparisons. *p < 0.05, **p < 0.01, ***p < 0.001

Morris water maze tests were conducted to examine whether the knockout of YKL-40 in astrocytes could rescue the memory deficits of 5xFAD mice. A significant decrease in escape latency over four days of training was defined as a strong learning ability in this spatial learning test. Memory deficits were defined by a reduced number of entries plus shortened latency to find the platform on day 5 (probe trial). No differences in escape latency between groups were observed on day 0 and day 1 (Fig. 6A, B). After four days of training, the escape latency of 5xFAD;Aldh;*Chi3l1*^fl/fl^ mice was significantly shortened compared with that of 5xFAD mice (Fig. 6C). As expected, WT, WT;Aldh;*Chi3l1*^fl/fl^, and 5xFAD;Aldh;*Chi3l1*^fl/fl^ mice showed greater decreases in escape latencies over four days of training ( Fig. 6C,I). On day 5, mice were allowed to explore the tank for 60 s following release from a fixed location (Fig. 6H). No differences in the total distance traveled and mean velocity were detected among the six groups (Fig. 6D, E). Deletion of YKL-40 increased the number of entries and reduced the escape latency of 5xFAD mice (Fig. 6F, G). No differences were observed between WT and WT;Aldh;*Chi3l1*^fl/fl^ mice (Fig. 6F, G). Taken together, the observed improvements in spatial memory and learning ability of YKL-40 knockout 5xFAD mice support the detrimental effects of YKL-40 in the Aβ deposition process.

### YKL-40 knockout enhances endocytosis through acidification of lysozyme

**Fig. 7:**
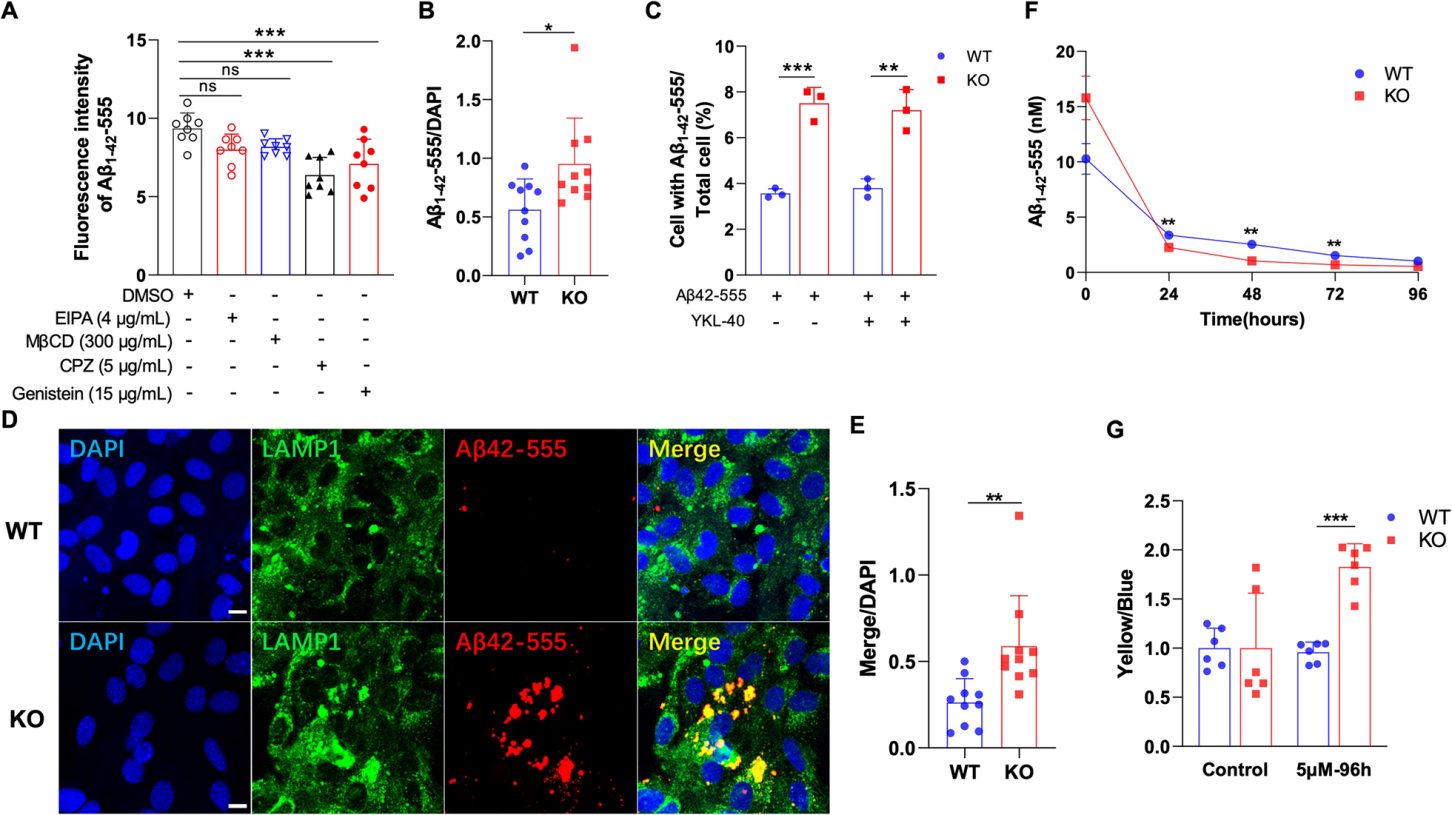
Knockout of YKL-40 enhances Aβ induced receptor mediated endocytosis and promotes Aβ lysosomal degradation in primary astrocytes. **A** Astrocytes were pre-treated with indicated endocytosis inhibitors for 6 h and then incubated with fluorescent labeled Aβ_1-42_ at a concentration of 0.1 µM for 24 h, the fluorescence intensity of Aβ_1-42_ up-taken by astrocytes was measured using plate reader (Bars, SD; n = 5; ns, not significant; ***p < 0.001).** B** Astrocytes were treated with fluorescent labeled Aβ_1-42_ (0.1 µM) for 24 h. The percentage of Aβ_1-42_ positive cells was analyzed using image J. Data are mean ± SD; n = 10, *p < 0.05. **C** Astrocytes were exposed fluorescent labeled Aβ_1-42_ (0.1 µM) together with or without recombinant YKL-40 protein (250 ng/mL) for 24 h, and the Aβ_1-42_ positive cells were analyzed with flow cytometry. Data are mean ± SD; n = 3; **p < 0.01, ***p < 0.001. **D** The sub-cellular localization of Aβ_42_-555 peptide in WT and YKL-40 KO astrocytes 24 h after addition was revealed by immunostaining with lysosomal protein LAMP1 (green signal), and Aβ_1-42_ (red signal). **E** Data are quantified as percentage of Aβ_1-42_ colocalized with lysosome was analyzed using image J. Data are mean ± SD; n = 10, **p < 0.01. **F** Astrocytes were pre-treated with fluorescent labeled Aβ_1-42_ (0.1 µM) for 24 h, and the levels of Aβ_1-42_ contained in cells after treatment were measured using plate reader every 24 h. Data are mean ± SD; n = 6, **p < 0.01.** G** Astrocytes were treated with Aβ_1-42_ (5 µM) for 96 h, the pH of lysosomes were measured using LysoSensor™ Yellow/Blue DND-160. Data are ± SD; n = 6, ***p < 0.001

To unravel the underlying mechanism responsible for the elevated Aβ plaque clearance following YKL-40 knockout, we compared the transcriptomes of primary astrocytes from WT and *Chi3l1*^−/−^ mice. Transcriptomic analysis revealed 294 up-regulated and 64 down-regulated genes with multiple enriched KEGG terms including phagosome (Additional file 1: Fig. S6A, B). GSEA analysis further identified genes involved in phagocytic vesicles and lysosome signaling (Additional file 1: Fig. S6C, D). We first tested if the endocytic pathway was required for Aβ uptake and retention in primary astrocytes using a panel of endocytic inhibitors. As shown in Fig. 7A, chlorpromazine (CPZ) and genistein, which inhibit clathrin-mediated and caveolae-mediated endocytosis, respectively, could significantly suppress the accumulation of fluorescence-labelled Aβ_1-42_–555 peptide. To examine if Aβ uptake was altered by YKL-40 knockout, primary astrocytes were exposed to Aβ_1-42_–555 peptide and the relative fraction of cells labelled positive were counted. There was a ~ twofold increase in Aβ_1-42_–555-positive cells in *Chi3l1*^−/−^ astrocytes compared to WT (Fig. 7B). Similar results were obtained by using the flow cytometry method (Fig. 7C). However, the addition of exogenous YKL-40 failed to restore homeostatic Aβ levels in *Chi3l1*^−/−^ cells suggesting that YKL-40 may act intracellularly (Fig. 7C). Furthermore, Aβ_1-42_ in *Chi3l1*^−/−^ cells was mainly localized in the lysosomes (Fig. 7D) and there was a ~ 2.5-fold more lysosomal-associated Aβ_1-42_ detected in *Chi3l1*^−/−^ than WT astrocytes (Fig. 7E). When lysosomal-associated Aβ_1-42_ was monitored over a period of 96 h, we observed that the rate of clearance was greater in *Chi3l1*^−/−^ astrocytes than in WT (Fig. 7F). We further monitored the lysosomal pH of astrocytes using LysoSensor dyes. While WT and *Chi3l1*^−/−^ astrocytes have similar lysosomal pH, treatment with Aβ_1-42_ for 96 h significantly increased the acidity of *Chi3l1*^−/−^ astrocytes by twofold when compared with WT (Fig. 7G). Taken together, these results imply that high level of YKL-40 in AD patients can promote neurotoxicity by acting directly at the level of Aβ uptake and degradation in astrocytes.

## Discussion

The present study revealed several major insights into the role of YKL-40 in AD. First, we demonstrated that YKL-40 levels in CSF are elevated with disease progression in 5xFAD mice. Second, YKL-40 could induce neurotoxicity, astrogliosis, microglia accumulation, and suppressed spontaneous electrical activities. More importantly, we have demonstrated drastic Aβ clearance in an astrocyte-specific knockout of YKL-40 and provided in vivo evidence of the rescue of memory deficits in this mouse line. Finally, our study established a transcriptional link between YKL-40 and the endocytic pathways, and thus providing a mechanistic basis for the rescued memory deficits seen in YKL-40 knockout mice.

Studies on YKL-40 have reported opposite biological functions in different disease pathologies. YKL-40 has been reported to be neuroprotective and/or anti-inflammatory in traumatic brain injury [38], experimental autoimmune encephalomyelitis [39], and bacterial infection [40]; whereas others have observed attenuated cell viability and retracted neurites in primary cortical neurons treated with YKL-40 [41, 42]. More consistent with the present study, it has been reported that a YKL-40 chemical inhibitor, K284-6111, could suppress Aβ deposition and restore memory function in 12 months old Tg2576 mice [43]. Similar to our findings, the therapeutic effects were immediately observed following a short treatment of only 28 days. The target for YKL-40 appears to be the ERK-dependent PTX3 pathway [43]. Separately, YKL-40 knockout in an APP/PS1 model was shown to impede amyloid accumulation [17]. They also demonstrated that YKL-40 was regulated by the circadian clock and could suppress the abilities of astrocytes or microglia to phagocytose Aβ. Similarly, our 5xFAD mouse model with the astrocyte-specific knockout of YKL-40 also demonstrated reduced amyloid plaque accumulation, reactive astrocytes, microglia and preserved synaptic structures. More critically, our study has gone further to demonstrate that YKL-40 knockout in astrocytes of 5xFAD mice rescued memory deficits.

The interaction between astrocytes and microglia plays a critical role in Aβ-induced neuroinflammation and plaque clearance. The study by Lananna *et. al.* indicated that YKL-40 knockout in APP/PS1 model did not alter the abundance of microglia cells but the levels of the lysosomal marker, CD68, were increased. In contrast, we observed a significant reduction in total IBA1-staining intensity in our knockout model to a level similar to wild-type mice. While we have not evaluated the functional states of microglia in our model, these differences may be due to the differences in the AD mouse models being used. Indeed, the ability of microglia cells to clear Aβ is still controversial and appears to be stage-specific [44–46].

The Morris water maze tests conducted using 7-month-old 5xFAD;Aldh;*Chi3l1*^fl/fl^ transgenic mice clearly demonstrated the rescue of cognitive impairment. Although the heterozygous loss of YKL-40 resulted in a 50% decrease in Aβ plaque deposition, no significant improvement in the Morris water maze test results was observed. The fact that YKL-40 was knockout just two weeks prior to the Morris water maze tests implies that symptomatic relief could be attainable even at the full onset of AD. This rapidity in plaque clearance and restoring memory functions suggests that YKL-40 may play a pivotal role in Aβ processing and clearance in 7-month-old 5XFAD mice. We also observed a greater number of NeuN + cells in the DG region of 5xFAD;Aldh;*Chi3l1*^fl/fl^ mice when compared to 5xFAD;Aldh;*Chi3l1*^+/+^ control. This is unlikely due to neurogenesis, but is more likely to be the result of reduced neuronal loss. Consistently, YKL-40 deletion moderately restored synaptophysin expression and p-AMAPR/AMPAR levels in 5xFAD;Aldh;*Chi3l1*^fl/fl^ mice compared with 5xFAD mice. Various studies have supported that decreased p-AMPAR Ser^845^ of GluA1 could lead to dysregulated synaptic plasticity and memory deficits. We speculate that the deletion of YKL-40 might restore GluA1 Ser^845^ phosphorylation and enhance AMPAR channel opening in 5xFAD;Aldh;*Chi3l1*^fl/fl^ mice. Because AMPAR function is highly related to long-term potentiation (LTP) [47, 48], future studies should investigate whether YKL-40/*Chi3l1-*induced AMPAR dysfunction and cognitive impairment are associated with altered LTP function.

Based on our RNA-Seq analysis, the observed enhanced clearance and degradation of Aβ in 5xFAD;Aldh;*Chi3l1*^fl/fl^ mice could be caused by the upregulation of genes involved in Aβ uptake and processing. For example, TLR2 has been shown to bind aggregated Aβ_42_ and inhibition of TLR2 in murine microglia abolished pro-inflammatory and phagocytotic responses following fibrillary Aβ exposure [49, 50]. Similarly, activation of TLR9 led to the amelioration of amyloid burden in the Tg2576 AD transgenic mouse model [51]. The greater rate of Aβ degradation observed in 5xFAD;Aldh;*Chi3l1*^fl/fl^ mice could be attributed to elevated expression of members of the cathepsin family of lysosomal proteases, including Cathepsin B, C, D, H, K, and S. Among them, cathepsin D (CTSD) mediates the degradation of both Aβ and tau in vitro and *Ctsd* knockout mice have 3- to fourfold increase in cerebral Aβ burden [52]. On the contrary, cathepsin S and B (CTSS and CTSB) are risk factor genes for AD as they produce toxic Aβ peptides through their β-secretase activities [53, 54]. Besides, the addition of exogenous YKL-40 to YKL-40 KO astrocytes did not reverse the enhanced Aβ uptake raising the possibility that either astrocytes lack YKL-40 receptors or the effect of YKL-40 on Aβ uptake acts through intracellular targets. Currently, there are 6 known receptors for YKL-40 including AGER, CD44, IL13RA2, LGALS3, PTGDR2, and TMEM219 [14]. A detailed expression analysis would be required to address the relative abundance of these receptors in mouse astrocytes. For potential intracellular targets for YKL-40, we have recently demonstrated the nuclear localization of YKL-40 in human glioblastoma cell lines [55]. Whether YKL-40 has direct transcriptional activity on gene expression could be an area of future investigation.

## Conclusion

The present study provides mechanistic insights supporting a functional role for YKL-40 in aggravating AD disease pathologies. There is strong evidence that elevated YKL-40 expression in CSF is associated with increasing Aβ burden. Extrapolate the data from astrocyte-specific knockout of YKL-40, it is concluded that YKL-40 is a risk gene for AD. First, it is toxic to neuronal cells by reducing cell viability, electrical activities, and dendritic processes. Second, it impedes Aβ uptake and subsequent degradation by astrocytes through transcriptional downregulation of genes involved in endocytosis, phagocytosis, and lysosomal functions. All these exacerbate the accumulation of Aβ in AD brains and provide credence for future development of therapeutics that target this molecule.

### Supplementary Information

**Additional file 1: Fig. S1.** Age-dependent amyloid-beta plaques deposition in 5xFAD mouse brains and Aβ-induced signaling in primary astrocytes. **A.** Confocal images (Mag. 10X) of stitched 4m (presymptomatic), 7m (symptomatic), and 9m (advanced) 5xFAD mouse brains immuno-stained with DAPI (blue signal), anti-Aβ (green signal), and anti-GFAP (red signal) antibodies. Scale bars, 2000 μm. **B.** Higher magnifications of dentate gyrus (DG) region of 7m brains are shown. **C.** The mean plaque surface area and plaque numbers in DG region were quantified using ImageJ software. 4m (n=8), 7m (n=8), 9m (n=6). Data are mean ± SEM. One-way ANOVA with Tukey’s post hoc comparisons.  *p < 0.05, **p < 0.01,***p < 0.001. **D.** Parallel immunofluorescence analysis similar to panel *(B)* was performed with Thioflavin T (ThT, green) and anti-YKL-40 (red). **E.** Quantification of data from Fig. 1J showing relative phosphorylation levels of AKT, P70S6, mTOR, and S6 following Aβ treatment. Data are mean ± SEM. One-way ANOVA with Tukey’s post hoc comparisons. ns, not significant, *p < 0.05, **p < 0.01. **Fig. S2.** Neuronal damages induced by Aβ_1-42_ (Aβ) and YKL-40 treatment. Primary neurons were derived from E17.5 wild type embryos. **A. **Brightfield photomicrographs taken from DIV14 treated with 10 μM Aβ, 400 ng/ml YKL-40, and 10 μM Aβ+400 ng/ml YKL-40 for 72 hrs. Scale bars, 200 μm. **B.** Binary figures of MAP2 immuno-stained confocal images, and their reconstructed Simple Neurite Tracing (SNT, Plugin in ImageJ software) tracing images following a 72 hrs exposure to the indicated concentrations of Aβ and YKL-40. **C**-**H.** Sholl Analysis (plugin from ImageJ) was performed from tracing images. Quantitative data for soma perimeter, dendritic length, axon length and nodes, dendritic nodes and ends are shown. Data are mean ± SEM of 50 to 70 neurons. *One-way ANOVA *with *Tukey’s post hoc *comparisons.  ***p < 0.001 compared to control. **Fig. S3.** Generation of astrocyte-specific YKL-40/*Chi3l1 *conditional knockout mice.** A.** Genotyping of Aldhl11-cre/ERT2. Tails of one month old pups from a typical litter were processed for DNA extraction and PCR analysis was carried out using the following primer pairs: *Aldhl1 *and *Aldhl2*; *Aldhl3* and *Aldhl4*. **B. **Knockout strategy of YKL-40/*Chi3l1*. **C.** Mating strategy used to generate the six experimental groups for this study. See Materials and Methods section for detailed descriptions. **D.** Tails of one month old pups from a typical litter were processed for DNA extraction and PCR analysis was carried out using the following primer pair: *mChil1_flox_F* and *mChil1_flox_R*. Wild-type (+/+) and heterozygous (+/- or +/fl) littermates are indicated. Abbreviations, Ma, markers; C, control. **E.** Primary astrocytes were treated without (-) or with (+) 1 μM tamoxifen solution**. **The 546-basepair (bp) knockout specific PCR product is indicated.** F.** Confocal images (Mag. 10X) of dentate gyrus (DG) and (**G**) thalamus (TH) regions of 3.5-month-old Aldh;*Chi3l1*^+/+^, Aldh;*Chi3l1*^+/fl^, and Aldh;*Chi3l1*^fl/fl^ mouse brains immuno-stained with DAPI (blue signal), anti-YKL-40 (green signal), and anti-GFAP (red signal) antibodies. Scale bars, 100 μm. All mice were IP injected with 20 mg/ml tamoxifen in corn oil (100 μl per mice/per day) for 7 consecutive days before brains were harvested. **Fig. S4.** Knockout of YKL-40 in astrocytes reduces Aβ deposition in 5xFAD mice brain. All mice were IP injected with 20 mg/ml tamoxifen in corn oil (100 µl per mice/per day) for 7 consecutive days before experiments. Experiments were carried out 7 days after last tamoxifen injection. **A.** Confocal images (Mag. 10X) of motor cortex + metrosplenial cortex (MOC), mortex (CTX), Cornu Ammonis 3 (CA3), and thalamus (TH) regions of 5xFAD;Aldh;*Chi3l1*^+/+^; 5xFAD;Aldh;*Chi3l1*^+/fl^; 5xFAD;Aldh;*Chi3l1*^fl/fl^ mouse brain immuno-stained with DAPI (blue signal), anti-Aβ (green signal), and anti-GFAP (red signal) antibodies. Scale bars, 100 μm. **B.** Quantification of plaque number from Aβ signals and mean surface area. Data are mean ± SEM. *One-way ANOVA* with *Tukey’s post hoc* comparisons. *p < 0.05,**p < 0.01, ***p < 0.001 compared to 5xFAD;Aldh;*Chi3l1*^+/+^. **Fig. S5.** YKL-40 depletion in astrocytes reduces glial activation. All mice were IP injected with 20 mg/ml tamoxifen in corn oil (100 μl per mice/per day) for 7 consecutive days before experiments. Experiments were conducted 7 days after last tamoxifen injection**. A-B.** Confocal images (Mag. 10X) of 7m 5xFAD mice brain immuno-stained with DAPI (blue signal), anti-IBA1 (green signal), and anti-GFAP (red signal) antibodies. Scale bar, 100 μm.** C**–**D.** Mean surface area was quantified. n=2 to 4. Data are mean ± SEM. **E**–**F.** Western blotting analysis of GFAP, and IBA1 expression from cortex (Cor) and hippocampal (Hip) lysates. GAPDH was used as an internal control. **G**-**H. **Quantification results of Western blot analysis are shown. Data are mean ± SEM. *One-way ANOVA *with *Tukey’s post hoc *comparisons.*p < 0.05, **p < 0.01,***p < 0.001. **Fig. S6.** Transcriptomic analysis of YKL-40 knockout astrocytes. **(A)** The volcano plot shows the 258 differentially expressed genes (294 up-regulated and 64 down-regulated, Foldchange>2, p<0.05) between WT and *Chi3l1*^-/-^ (KO) in primary astrocytes by RNA-seq.  (**B**) KEGG enrichment analysis of all DEGs showed that KO-associated with phagosome signaling pathway. GSEA analysis showed that genes associated with **(C)** phagocytic vesicles and **(D) **lysosome signaling were significantly up-regulated in KO astrocytes.

## Data Availability

All data generated or analysed during this study are included in this published article [and its Additional files].

## Abbreviations

Aβ: Amyloid-beta
AD: Alzheimer’s disease
BACE1: β-Site amyloid precursor protein cleaving enzyme 1
CA3: Cornu Ammonis 3
CSF: Cerebrospinal fluid
*Chi3l1*: Chitinase-3 like 1
CTX: Cortex
DIV: Days in vitro
DG: Dentatae gyrus
MOC: Motor cortex + retrosplenial cortex
TH: Thalamus
MEA: Microelectrode array
PI3-K: Phosphoinositide 3-kinase
PDL: Poly-D-lysine; and S6K, S6 kinase
