# Supplementary material for: Astrocyte-specific knockout of YKL-40/Chi3l1 reduces Aβ burden and restores memory functions in 5xFAD mice
Source: J Neuroinflammation. 2023 Dec 2;20:290. doi: 10.1186/s12974-023-02970-z (PMC10693711; doi:10.1186/s12974-023-02970-z)
Supplement: Supplementary file 1 — Additional file 1: Fig. S1. Age-dependent amyloid-beta plaques deposition in 5xFAD mouse brains and Aβ-induced signaling in primary astrocytes. A. Confocal images (Mag. 10X) of stitched 4m (presymptomatic), 7m (symptomatic), and 9m (advanced) 5xFAD mouse brains immuno-stained with DAPI (blue signal), anti-Aβ (green signal), and anti-GFAP (red signal) antibodies. Scale bars, 2000 μm. B. Higher magnifications of dentate gyrus (DG) region of 7m brains are shown. C. The mean plaque surface area and plaque numbers in DG region were quantified using ImageJ software. 4m (n=8), 7m (n=8), 9m (n=6). Data are mean ± SEM. One-way ANOVA with Tukey’s post hoc comparisons. *p < 0.05, **p < 0.01,***p < 0.001. D. Parallel immunofluorescence analysis similar to panel (B) was performed with Thioflavin T (ThT, green) and anti-YKL-40 (red). E. Quantification of data from Fig. 1J showing relative phosphorylation levels of AKT, P70S6, mTOR, and S6 following Aβ treatment. Data are mean ± SEM. One-way ANOVA with Tukey’s post hoc comparisons. ns, not significant, *p < 0.05, **p < 0.01. Fig. S2. Neuronal damages induced by Aβ1-42 (Aβ) and YKL-40 treatment. Primary neurons were derived from E17.5 wild type embryos. A. Brightfield photomicrographs taken from DIV14 treated with 10 μM Aβ, 400 ng/ml YKL-40, and 10 μM Aβ+400 ng/ml YKL-40 for 72 hrs. Scale bars, 200 μm. B. Binary figures of MAP2 immuno-stained confocal images, and their reconstructed Simple Neurite Tracing (SNT, Plugin in ImageJ software) tracing images following a 72 hrs exposure to the indicated concentrations of Aβ and YKL-40. C-H. Sholl Analysis (plugin from ImageJ) was performed from tracing images. Quantitative data for soma perimeter, dendritic length, axon length and nodes, dendritic nodes and ends are shown. Data are mean ± SEM of 50 to 70 neurons. One-way ANOVA with Tukey’s post hoc comparisons. ***p < 0.001 compared to control. Fig. S3. Generation of astrocyte-specific YKL-40/Chi3l1 conditional knockout mice. A. Genotypin [file 12974_2023_2970_MOESM1_ESM.docx]

**Supporting Information for**

**Astrocyte-specific knockout of YKL-40/*Chi3l1* reduces Aβ burden and restores memory functions in 5xFAD mice**

Xiaoyan Zeng, Stanley K.K. Cheung, Mengqi Shi, Penelope M.Y. Or, Zhining Li, Julia Y.H. Liu, Wayne L.H. Ho, Tian Liu, Kun Lu, John A. Rudd, Yubing Wang, and Andrew M. Chan

**This PDF file includes:**

Supporting Materials and Methods

Figures S1 to S6

**Supplemental Materials and Methods**

Antibodies

| **Antibodies** | **Manufacture** | **Cat#** |
| --- | --- | --- |
| AKT | Cell Signaling | 9272 |
| Alexa Fluor 488 Goat-anti-mouse secondary | Thermo Fisher Scientific | A11001 |
| Alexa Fluor 488 Goat-anti-rabbit secondary | Thermo Fisher Scientific | A11008 |
| Alexa Fluor 555 Goat-anti-chicken secondary | Thermo Fisher Scientific | A21437 |
| Alexa Fluor 555 Goat-anti-mouse secondary | Thermo Fisher Scientific | A21424 |
| Alexa Fluor 647 Goat-anti-rat secondary | Thermo Fisher Scientific | A21434 |
| AMPAR | Cell Signaling | 13185S |
| Anti-goat HRP-conjugate secondary | Santa Cruz | SC2352 |
| Anti-mouse HRP-conjugated secondary | Cell Signaling | 7076s |
| Anti-rabbit HRP-conjugated secondary | Cell Signaling | 7074P2 |
| Anti-rat HRP-conjugated secondary | Cell Signaling | 7077 |
| Aβ | Cell Signaling | 8243 |
| GAPDH | Cell Signaling | 5174 |
| GFAP | Cell Signaling | 3670 |
| GFAP CY3 | Sigma | C9205 |
| IBA1 | Wako | 019-19741 |
| JAK2 | Cell Signaling | 3230 |
| LAMP1 | Abcam | Ab208943 |
| MAP2 | Abcam | ab5392 |
| mTOR | Cell Signaling | 2983 |
| NEUN | Abcam | ab177487 |
| NEUN Alexa Fluor 488 | Millipore | ABN78A4 |
| p-AKT Thr308 | Cell Signaling | 9275 |
| p-AKT Ser473 | Cell Signaling | 4060 |
| p-AMPAR Ser845 | Cell Signaling | 8084 |
| p-JAK2 Tyr1007/1008 | Cell Signaling | 3776 |
| p-mTOR Ser2448 | Cell Signaling | 5536 |
| p-p70 S6K Thr389 | Cell Signaling | 9206 |
| p-S6 Ser235/236 | Cell Signaling | 4858 |
| p-STAT3 Tyr705 | Cell Signaling | 9145 |
| p-STAT3 Tyr705 | Cell Signaling | 9138 |
| p70 S6K | Cell Signaling | 9202 |
| PSD-95 | Abcam | ab18258 |
| S6 | Cell Signaling | 2317 |
| STAT3 | Cell Signaling | 12640 |
| Synapsin-1 | Cell Signaling | 5297 |
| Synaptophysin (SYP) | Millipore | S5768 |
| Synaptophysin (SYP) | Cell Signaling | 5461 |
| YKL-40 | R & D | MAB2649 |
| YKL-40 | Invitrogen | PA5-43746 |
| β-Actin | Cell Signaling | 3700 |
| β-Actin | Cell Signaling | 4967 |

Genotyping

All genotyping protocols were provided by either the Jackson Laboratory or Cyagen, Inc. The primers used for each mouse strain are as follows:

5xFAD: APP1+APP2; PSEN1+PSEN2

Chi3l1fl: mChil1_flox_F+mChil1_flox_R

Aldh-Cre: Aldh1+Aldh2; Aldh3+Aldh4

Primers

| **Primers for genotyping** |  |
| --- | --- |
| *APP1* | AGGACTGACCACTCGACCAG |
| *APP2* | CGGGGGTCTAGTTCTGCAT |
| *PSEN1* | AATAGAGAACGGCAGGAGCA |
| *PSEN2* | GCCATGAGGGCACTAATCAT |
| *mChil1_flox_F* | GCAGAGAGATGGTGTAAACAGACT |
| *mChil1_flox_R* | TTAAACCCAGTGCACACCCATTA |
| *Aldh1l1* | CTGTCCCTGTATGCCTCTGG |
| *Aldh1l2* | AGATGGAGAAAGGACTAGGCTACA |
| *Aldh1l3* | GGCAAACGGACAGAAGCA |
| *Aldh1l4* | CTTCAACAGGTGCCTTCCA |

Code for Spike Analyze

clear all

clc

rng(1);

warning('off','signal:findpeaks:largeMinPeakHeight')

warning('off','MATLAB:xlswrite:AddSheet')

import McsHDF5.*

%% User select files to analyze

[file,path] = uigetfile('*.h5'); % Select .h5 file

folderex = uigetdir; % Select Export folder

%% User input threshold value

prompt = {'Threshold (uV) (No need to enter minus sign','Average noise level (uV)','Apply filter? yes=1/no=2'};

dlgtitle = 'Input Panel';

dims = [1 35];

definput = {'80','40','2'};

answer1 = inputdlg(prompt,dlgtitle,dims,definput);

datafile = McsHDF5.McsData(append(path,file));

ch = 60; % number of channel is 60

sf = 25000; % Sampling frequency

fileA = zeros(ch,length(datafile.Recording{1}.AnalogStream{1}.ChannelData(1,:)));

for b = 1:ch

fileA(b,:) = datafile.Recording{1}.AnalogStream{1}.ChannelData(b,:);

end

l = size(fileA,2);

data_unflit = (fileA/1000000)'; % the data in .h5 is somehow X 1000000 in unit

%% Data filtering

if str2double(cell2mat(answer1(3))) == 1

Fn = sf/2; % Nyquist Frequency

Wp = [100 4000]/Fn; % Passband Normalised

Ws = [50 6000]/Fn; % Stopband Normalised % original [0.01 1.4]

Rp = 1; % Passband Ripple (Irrelevant in Butterworth)

Rs = 50; % Stopband Attenuation

[n,Wp] = ellipord(Wp,Ws,Rp,Rs); % Order Calculation

[z,p,k] = ellip(n,Rp,Rs,Wp); % Zero-Pole-Gain

[sos,g] = zp2sos(z,p,k); % Second-Order Section For Stability

data = zeros(l,ch);

disp('Filtering data...')

for i = 1:ch

data(:,i) = filtfilt(sos, g, data_unflit(:,i));

end

end

% Check

%figure

%plot(data_unflit(:,39))

%hold on

%plot(data(:,39),'r')

%xlim([1299820 1948900])

%% Channel Convertion from Channel 1-60 to Corresponding electrodes

% Reorganize position

chnum = [11 12 13 14 15 16 17 18 21 22 23 24 25 26 27 28 31 32 33 34 35 36 37 38 41 42 43 44 45 46 47 48 51 52 53 54 55 56 57 58 61 62 63 64 65 66 67 68 71 72 73 74 75 76 77 78 81 82 83 84 85 86 87 88];

% for .h5 files

order = [61 21 19 16 15 12 10 61 24 22 20 17 14 11 9 7 26 25 23 18 13 8 6 5 29 30 28 27 4 3 1 2 32 31 33 34 57 58 60 59 35 36 38 43 48 53 55 56 37 39 41 44 47 50 52 54 61 40 42 45 46 49 51 61];

if str2double(cell2mat(answer1(3))) == 1

data(:,61) = NaN;

data_order = zeros(size(data,1),ch+4);

for io = 1:ch+4

data_order(:,io) = data(:,order(io));

end

else

data_unflit(:,61) = NaN;

data_order = zeros(size(data_unflit,1),ch+4);

for io = 1:ch+4

data_order(:,io) = data_unflit(:,order(io));

end

end

%% Analyze %%%%%%%%%%%%%%%%%%%%%%%%%%%%%%%%%%%%%%%%%%%%%%%%%%

B = 1;

for j = 1:ch+4

clear h1 h2

% Peak find

d = data_order(:,j);

dLen = length(d);

[pk,t] = findpeaks(-d,'MinPeakHeight',str2double(cell2mat(answer1(1))));

Spkfind = zeros(126,size(t,1)); % 5000 +7500 +1

Spktime = 0;

% Plot spikes

for i = 1:size(t,1)

st = max(t(i,1) - 50,1); % starting time and ending time of spike to be plotted

ed = min(t(i,1) + 75,dLen);

if t(i,1) - 50 < 1

Spkfind(50-t(i,1)+2:126,i) = d(st:ed)

elseif t(i,1) + 75 > dLen

Spkfind(1:ed-st+1,i) = d(st:ed);

else

Spkfind(:,i) = d(st:ed);

end

Spktime(i,1) = t(i);

%plot(d(st:ed),'b')

%hold on

end

%xlabel('Time(s)');ylabel('Voltage(\muV)');

%hold off

% Filter wrongly identified spikes

Spkfind_2 = NaN(126,1); Spktime_2 = 0;

AA = 1;

for ff = 1:size(Spkfind,2)

if mean(Spkfind(1:30,ff)) < -str2double(cell2mat(answer1(2)))

elseif mean(Spkfind(96:126,ff)) < -str2double(cell2mat(answer1(2)))

else

Spkfind_2(:,AA) = Spkfind(:,ff);

Spktime_2(AA,1) = Spktime(ff,1);

AA = AA + 1;

end

end

a = NaN(size(Spkfind_2,2),6);

if size(Spkfind_2,2) == 0

elseif size(Spkfind_2,2) == 1

else

for i = 1:size(Spkfind_2,2)

dd = Spkfind_2(:,i);

e = findchangepts(dd,'MaxNumChanges',2); % find turning points

if sum(size(e)) == 2 % set NaN to those spike which cannot idfy tp

e(1,1) = NaN; e(2,1) = NaN; p = NaN; auc = NaN;

elseif size(e,1) == 0

e(1,1) = NaN; e(2,1) = NaN; p = NaN; auc = NaN;

else

p = (e(2,1)-e(1,1))/sf; % find period of spike

auc = trapz(dd(e(1,1):e(2,1))); % find area under curve within spike

end

[mi,~] = min(dd); % find amplitude

[sl,~] = min(diff(dd)); % find negative slope

a(i,1) = mi; a(i,2) = (e(1,1)-50)/sf; a(i,3) = (e(2,1)-50)/sf; a(i,4) = p; a(i,5) = sl; a(i,6) = auc;

end

end

% Filter away Spkfind without turning point

Spkfind_3 = NaN(126,1); Spktime_3 = 0;

a_2 = NaN(1,6);

A = 1;

for k = 1:size(a,1)

if isnan(a(k,2))

else

Spkfind_3(:,A) = Spkfind_2(:,k);

Spktime_3(A,1) = Spktime_2(k,1);

a_2(A,:) = a(k,:);

A = A + 1;

end

end

if size(Spkfind_3,2) == 0

elseif size(Spkfind_3,2) == 1

else

h1 = figure ('visible','off');

scatter(a_2(:,1),a_2(:,6),'filled')

[idx,cen] = kmeans(a_2(:,[1 6]),2); % kmeans clustering to identify no. of different spike shapes

hold on

%scatter(cen(:,1),cen(:,2),'filled','SizeData',5000,'MarkerFaceColor','r','MarkerFaceAlpha',0.5)

plot(cen(1:2,1),cen(1:2,2),'r.','MarkerSize',30,'LineStyle','none');

title(chnum(j));

hold off

xlabel('Amplitude(\muV)');ylabel('Area under curve');

idx2 = idx';

h2 = figure ('visible','off');

for i = 1:size(a_2,1)

curTime = NaN(126,1)

for cnt = 1:126

curTime(cnt,1) = cnt*0.04

end

if idx2(1,i) == 1

plot(curTime, Spkfind_3(:,i),'b')

elseif idx2(1,i) == 2

plot(curTime, Spkfind_3(:,i),'g')

elseif idx2(1,i) == 3

plot(curTime, Spkfind_3(:,i),'r') % Max no. of spike shape limited to 3

else

end

hold on

end

%set(gca,'XTick',[], 'YTick', []), xlim([0 126]) ,title(chnum(j));

title(chnum(j));

xlabel('Time(ms)');ylabel('Voltage(\muV)');

hold off

end

if size(Spkfind,2) == 0

elseif size(Spkfind,2) == 1

else

try

saveas(h1,fullfile(folderex,['\' num2str(chnum(j)) '_scatter' '.png']));

saveas(h2,fullfile(folderex,['\' num2str(chnum(j)) '_spikesort' '.png']));

catch

end

end

% Combine all spikes in one sheet

for cc = 1:size(Spkfind_3,2)

Channum(:,B) = chnum(j);

Comspike(:,B) = Spkfind_3(:,cc);

Comdata(B,:) = a_2(cc,:);

Comtime(B,1) = Spktime_3(cc,1);

B = B + 1;

end

% Output

if size(Spkfind,2) == 0

elseif size(Spkfind,2) == 1

else

Out1lb = [{"Amphitude (uV)","TP1 (s)","TP2 (s)","Period (s)","Slope (uV/s)","AUC","Time"}];

Out1full = [Out1lb; num2cell(a_2) num2cell(Spktime_3/sf)];

OutSpiketime = [Spktime_3/sf Spkfind_3'];

xlswrite(fullfile(folderex,['\Output.xlsx']),Out1full,num2str(chnum(j)))

xlswrite(fullfile(folderex,['\Spikes.xlsx']),OutSpiketime,num2str(chnum(j)))

end

disp(['Finish channel: ' num2str(chnum(j))])

end

return

%% PART 2 - Clustering spikes and Export Full Summary

%% Select the Appropriate number of cluster

%% Copy and Paste the following codes to command and press enter

%%%%%%%%%%%%%%%%%%%%%%%%%%%BELOW THIS LINE%%%%%%%%%%%%%%%%%%%%%%%%%%%%%%%%%%

prompt = {'Clusters (Max 5)'}; % User input number of clusters (Maximum 5)

dlgtitle = 'Input Panel';

dims = [1 35];

definput = {'5'};

answer2 = inputdlg(prompt,dlgtitle,dims,definput);

clear idx3 cen

% Clustering final spikes into groups

[idx3,cen3] = kmeans(Comdata(:,[1 6]),str2double(cell2mat(answer2(1)))); % kmeans clustering to identify no. of different spike shapes

h3 = figure ('visible','off');

hold on

for bbb = 1:size(Comdata,1)

if idx3(bbb,1) == 1

scatter(Comdata(bbb,1),Comdata(bbb,6),'filled','b')

elseif idx3(bbb,1) == 2

scatter(Comdata(bbb,1),Comdata(bbb,6),'filled','g')

elseif idx3(bbb,1) == 3

scatter(Comdata(bbb,1),Comdata(bbb,6),'filled','r')

elseif idx3(bbb,1) == 4

scatter(Comdata(bbb,1),Comdata(bbb,6),'filled','m')

elseif idx3(bbb,1) == 5

scatter(Comdata(bbb,1),Comdata(bbb,6),'filled','c')

end

end

%scatter(Comdata(:,1),Comdata(:,6),'filled','b')

%scatter(cen(:,1),cen(:,2),'filled','SizeData',5000,'MarkerFaceColor','r','MarkerFaceAlpha',0.5)

plot(cen3(:,1),cen3(:,2),'k.','MarkerSize',30,'LineStyle','none');

for ccc = 1:size(cen3,1)

text(cen3(ccc,1)+4,cen3(ccc,2),['\color{black}\fontsize{14}' num2str(ccc)])

end

title('All spikes');

xlabel('Amplitude(\muV)'); ylabel('Area under curve');

hold off

idx4 = idx3';

h4 = figure ('visible','off');

hold on

for i = 1:size(Comdata,1)

if idx4(1,i) == 1

plot(Comspike(:,i),'b')

elseif idx4(1,i) == 2

plot(Comspike(:,i),'g')

elseif idx4(1,i) == 3

plot(Comspike(:,i),'r')

elseif idx4(1,i) == 4

plot(Comspike(:,i),'m')

elseif idx4(1,i) == 5

plot(Comspike(:,i),'c') % Max no. of spike shape limited to 3

else

end

end

title('All spikes');

xlabel('Time(s)');ylabel('Voltage(\muV)');

hold off

saveas(h3,fullfile(folderex,['\All_scatter.png']));

saveas(h4,fullfile(folderex,['\All_spikesort.png']));

% Combine final output

Out2lb = [{"CH","Group","Time","Amphitude (uV)","TP1 (s)","TP2 (s)","Period (s)","Slope (uV/s)","AUC"}];

Out2full = [Out2lb; num2cell(Channum)' num2cell(idx3) num2cell(Comtime/sf) num2cell(Comdata)];

Out3full = [num2cell(Channum)' num2cell(idx3) num2cell(Comtime/sf) num2cell(Comspike)'];

xlswrite(fullfile(folderex,['\Summary.xlsx']),Out2full,'summary')

xlswrite(fullfile(folderex,['\Summary.xlsx']),Out3full,'allspikes')

disp('Ignore the warning and Completed')

%%%%%%%%%%%%%%%%%%%%%%%%%%ABOVE THIS LINE%%%%%%%%%%%%%%%%%%%%%%%%%%%%%%%%%%

return

%% PART 3 ======= Raw trace of one selected channel

%%%%%%%%%%%%%%%%%%%%%%%%%%%%%%%%%%%%%%%%%%%%%%%%%%%%%%%%%%%%%%%%%%%%%%%%%%%

prompt = {'Which channel?'}; % User input number of clusters (Maximum 5)

dlgtitle = 'Input Panel';

dims = [1 35];

definput = {'77'};

answer3 = inputdlg(prompt,dlgtitle,dims,definput);

idx5 = find(chnum==str2double(cell2mat(answer3(1))));

figure

hold on

plot(data_unflit(:,order(idx5)),'k')

xlabel('Time (s)');ylabel('Voltage (\muV)');

xt = get(gca, 'XTick');

set(gcf,'position',[800,100,600,200])

hold off

%%%%%%%%%%%%%%%%%%%%%%%%%%%%%%%%%%%%%%%%%%%%%%%%%%%%%%%%%%%%%%%%%%%%%%%%%%%

return

%% PART 4 ===== Raster Plot of several selected channels

%%%%%%%%%MUST OUTPUT THE FULL SUMMARY BEFORE RUNNING THE FOLLOWING%%%%%%%%%

prompt = {'Which channel to be drawn on Raster Plot?(Use commas to seperate each channels'};

dlgtitle = 'Input Panel';

dims = [1 35];

%definput = {'16,25,33,34,46,56,65,66,77'};

definput = {'11,12,13,14,15,16,17,18,21,22,23,24,25,26,27,28,31,32,33,34,35,36,37,38,41,42,43,44,45,46,47,48,51,52,53,54,55,56,57,58,61,62,63,64,65,66,67,68,71,72,73,74,75,76,77,78,81,82,83,84,85,86,87,88'};

answer4 = inputdlg(prompt,dlgtitle,dims,definput);

anssep = strsplit(answer4{1},',');

anssep2 = cellfun(@str2double, anssep);

figure

hold on

for ia = 1:size(anssep2,2)

for ja = 1:size(Comtime,1)

if Channum(1,ja) == anssep2(ia)

if Comtime(ja,1) == 0

else

scatter(Comtime(ja,1),ia,25,'k','filled')

end

end

end

end

xlabel('Time (s)');ylabel('Channel number');

ylim([1 size(anssep2,2)])

set(gca, 'YTick', 1:size(anssep2,2))

set(gca, 'YTickLabel',anssep) % for 0.75

set(gcf,'position',[800,400,600,200]) %For single drug data

xt = get(gca, 'XTick');

set(gca, 'XTick',xt, 'XTickLabel',round(xt/25000,0))

hold off

%%%%%%%%%%%%%%%%%%%%%%%%%%%%%%%%%%%%%%%%%%%%%%%%%%%%%%%%%%%%%%%%%%%%%%%%%%%


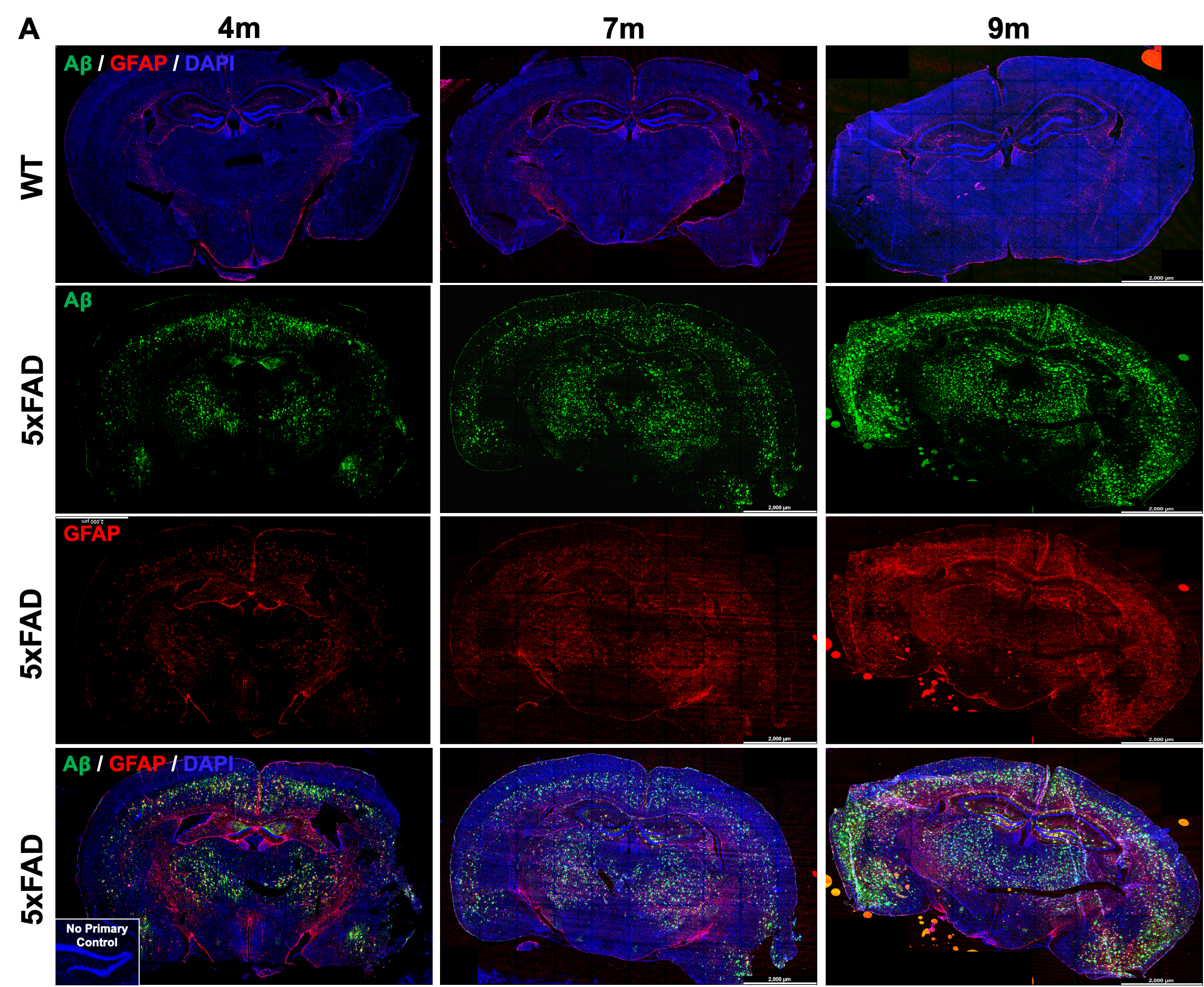


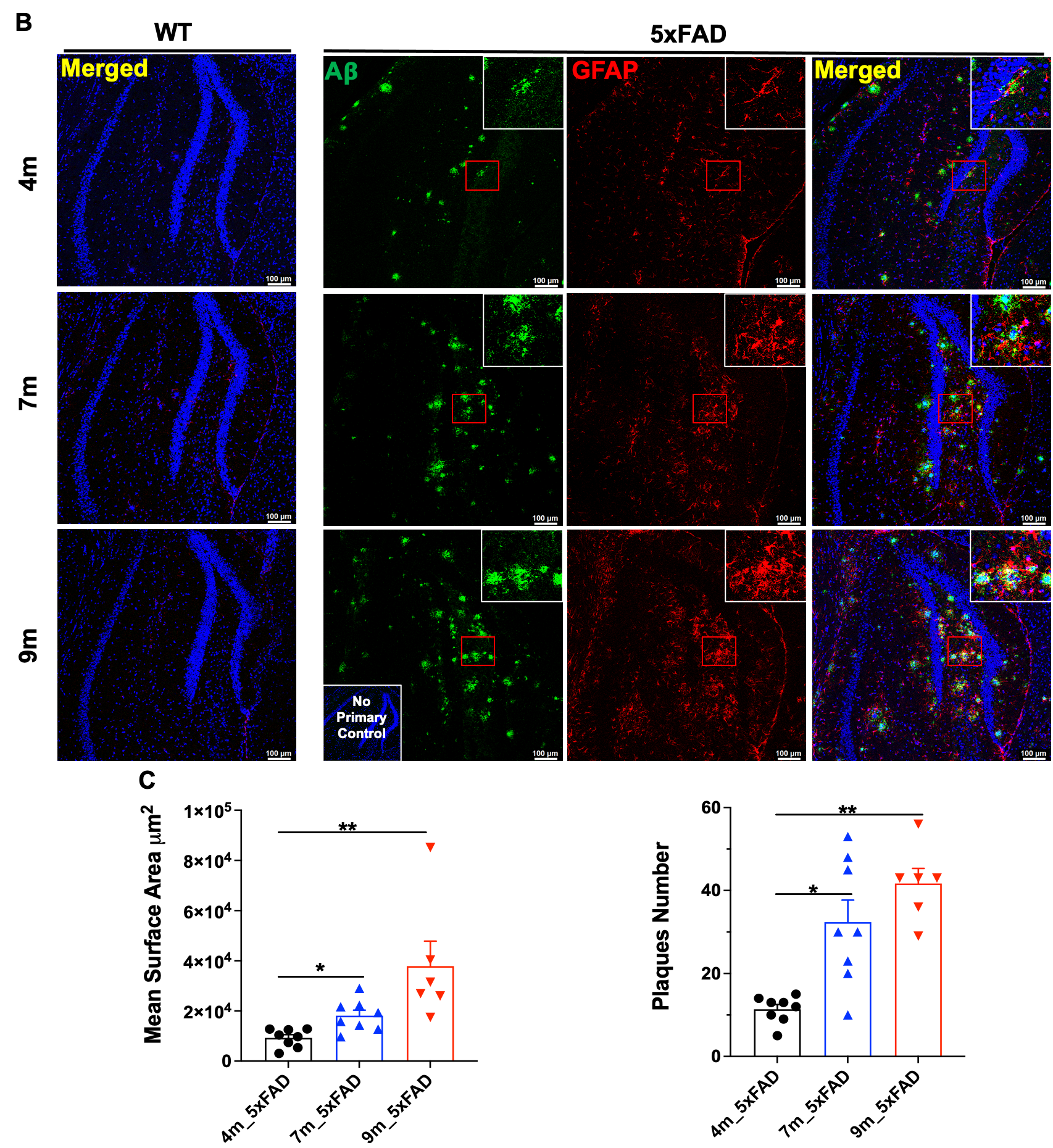


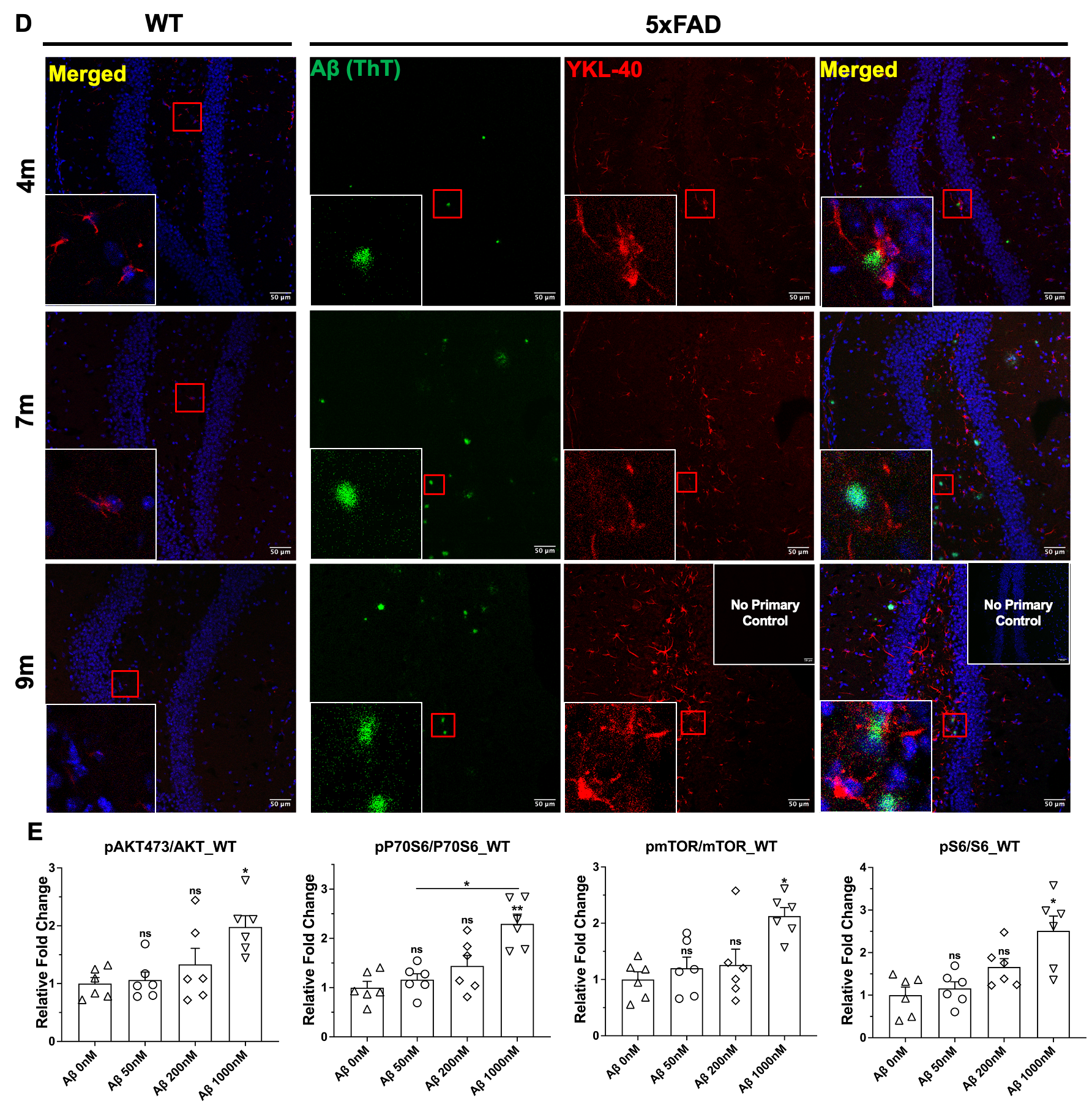


**Fig. S1. Age-dependent amyloid-beta plaques deposition in 5xFAD mouse brains and Aβ-induced signaling in primary astrocytes**. **A.** Confocal images (Mag. 10X) of stitched 4m (presymptomatic), 7m (symptomatic), and 9m (advanced) 5xFAD mouse brains immuno-stained with DAPI (blue signal), anti-Aβ (green signal), and anti-GFAP (red signal) antibodies. Scale bars, 2000 μm. **B.** Higher magnifications of dentate gyrus (DG) region of 7m brains are shown. **C.** The mean plaque surface area and plaque numbers in DG region were quantified using ImageJ software. 4m (n=8), 7m (n=8), 9m (n=6). Data are mean ± SEM. One-way ANOVA with Tukey’s post hoc comparisons. **p* < 0.05, ***p* < 0.01, ****p* < 0.001. **D.** Parallel immunofluorescence analysis similar to panel *(B)* was performed with Thioflavin T (ThT, green) and anti-YKL-40 (red). **E.** Quantification of data from Fig. 1J showing relative phosphorylation levels of AKT, P70S6, mTOR, and S6 following Aβ treatment. Data are mean ± SEM. One-way ANOVA with Tukey’s post hoc comparisons. ns, not significant, **p* < 0.05, ***p* < 0.01.


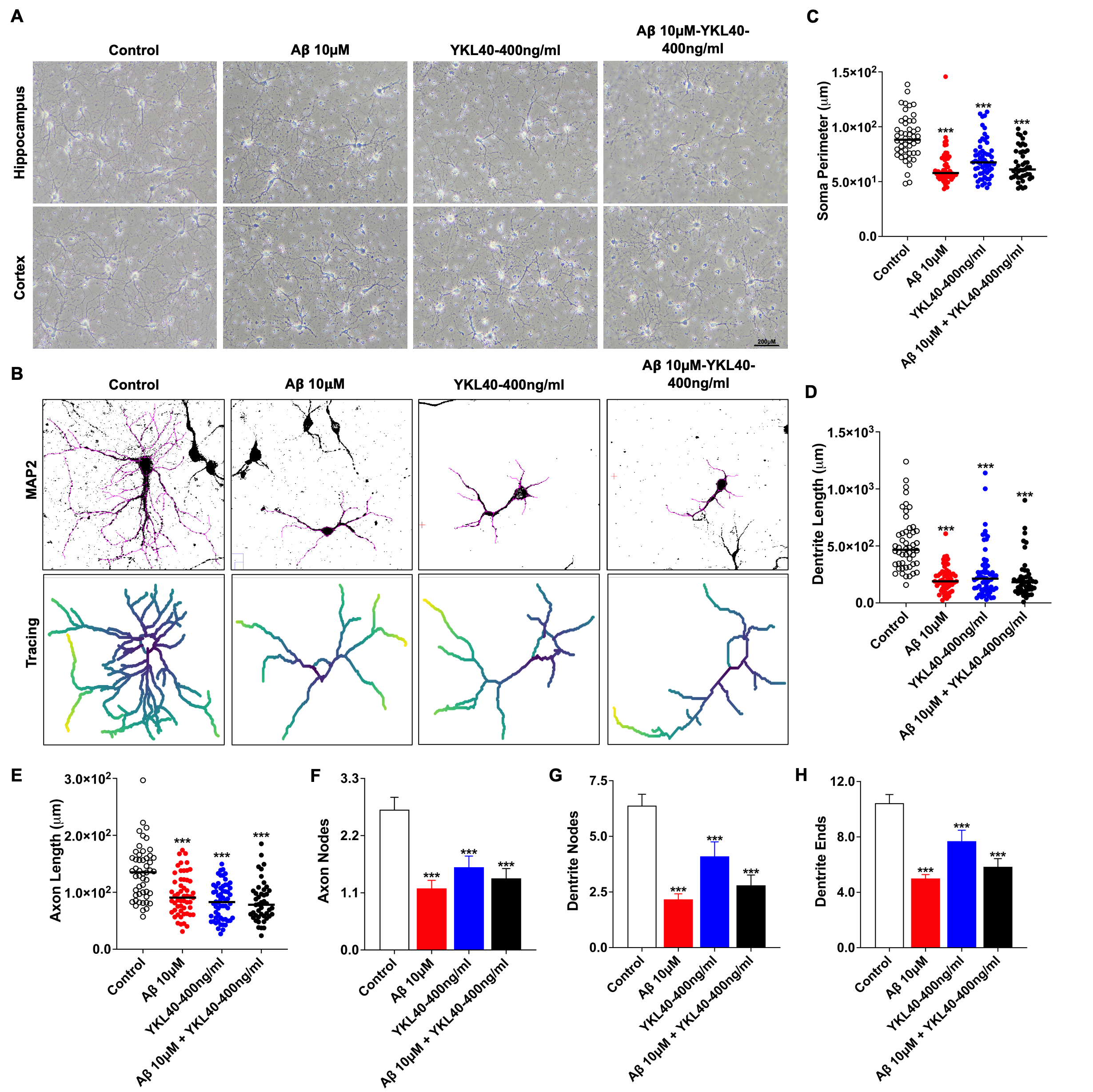


**Fig. S2. Neuronal damages induced by Aβ_1-42_ (Aβ) and YKL-40 treatment.** Primary neurons were derived from E17.5 wild type embryos. **A.** Brightfield photomicrographs taken from DIV14 treated with 10 μM Aβ, 400 ng/ml YKL-40, and 10 μM Aβ+400 ng/ml YKL-40 for 72 hrs. Scale bars, 200 μm. **B.** Binary figures of MAP2 immuno-stained confocal images, and their reconstructed Simple Neurite Tracing (SNT, Plugin in ImageJ software) tracing images following a 72 hrs exposure to the indicated concentrations of Aβ and YKL-40. **C-H.** Sholl Analysis (plugin from ImageJ) was performed from tracing images. Quantitative data for soma perimeter, dendritic length, axon length and nodes, dendritic nodes and ends are shown. Data are mean ± SEM of 50 to 70 neurons. *One-way ANOVA* with *Tukey’s post hoc* comparisons. ****p* < 0.001 compared to control.

**G**

**F**

**
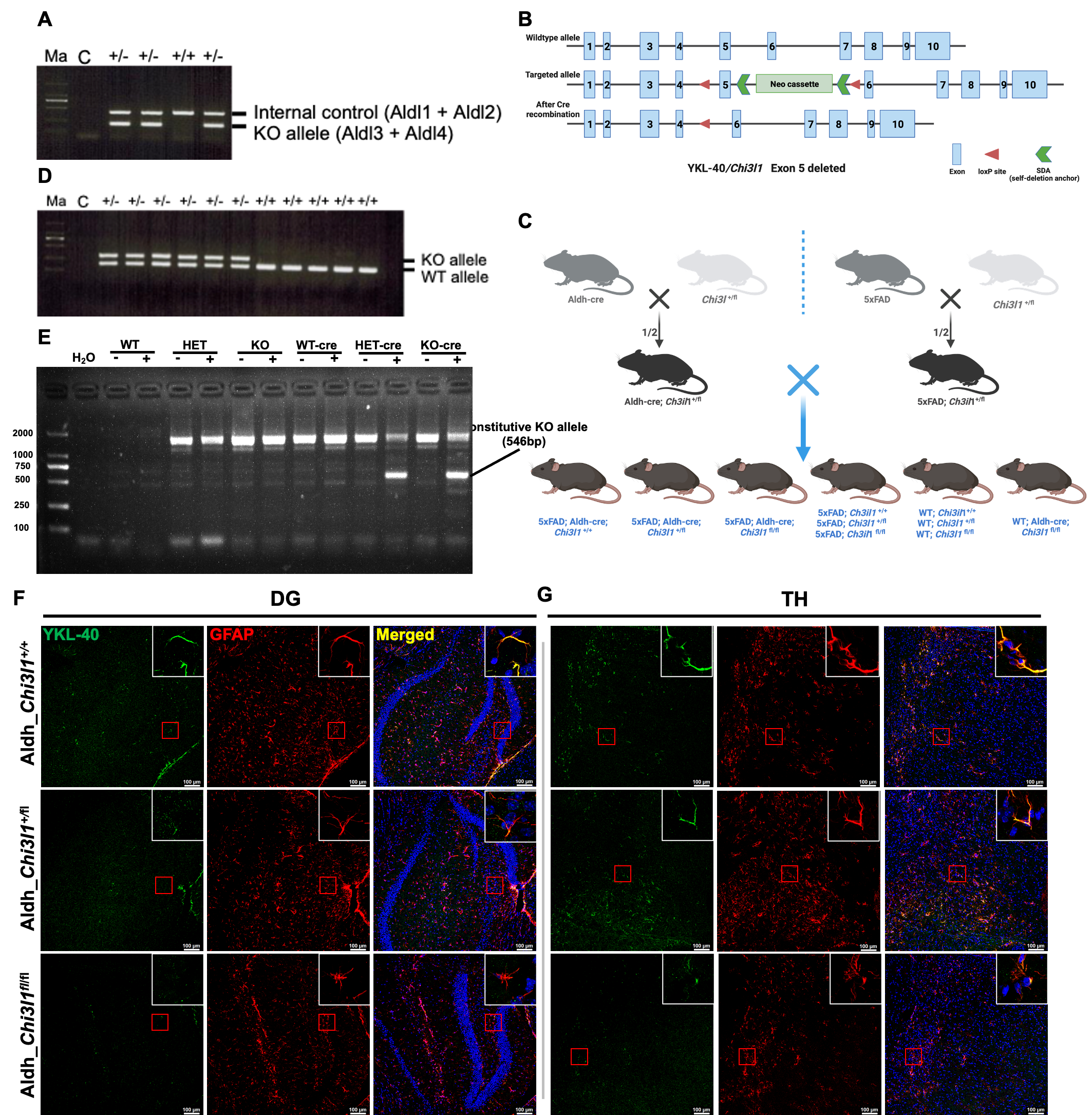
Fig. S3. Generation of astrocyte-specific YKL-40/*Chi3l1* conditional knockout mice. A.** Genotyping of Aldhl11-cre/ERT2. Tails of one month old pups from a typical litter were processed for DNA extraction and PCR analysis was carried out using the following primer pairs: *Aldhl1* and *Aldhl2*; *Aldhl3* and *Aldhl4*. **B.** Knockout strategy of YKL-40/*Chi3l1*. **C.** Mating strategy used to generate the six experimental groups for this study. See Materials and Methods section for detailed descriptions. **D.** Tails of one month old pups from a typical litter were processed for DNA extraction and PCR analysis was carried out using the following primer pair: *mChil1_flox_F* and *mChil1_flox_R*. Wild-type (+/+) and heterozygous (+/- or +/fl) littermates are indicated. Abbreviations, Ma, markers; C, control. **E.** Primary astrocytes were treated without (-) or with (+) 1 μM tamoxifen solution**.** The 546-basepair (bp) knockout specific PCR product is indicated. **F.** Confocal images (Mag. 10X) of dentate gyrus (DG) and **(G)** thalamus (TH) regions of 3.5-month-old Aldh;*Chi3l1*^+/+^, Aldh;*Chi3l1*^+/fl^, and Aldh;*Chi3l1*^fl/fl^ mouse brains immuno-stained with DAPI (blue signal), anti-YKL-40 (green signal), and anti-GFAP (red signal) antibodies. Scale bars, 100 μm. All mice were IP injected with 20 mg/ml tamoxifen in corn oil (100 μl per mice/per day) for 7 consecutive days before brains were harvested.


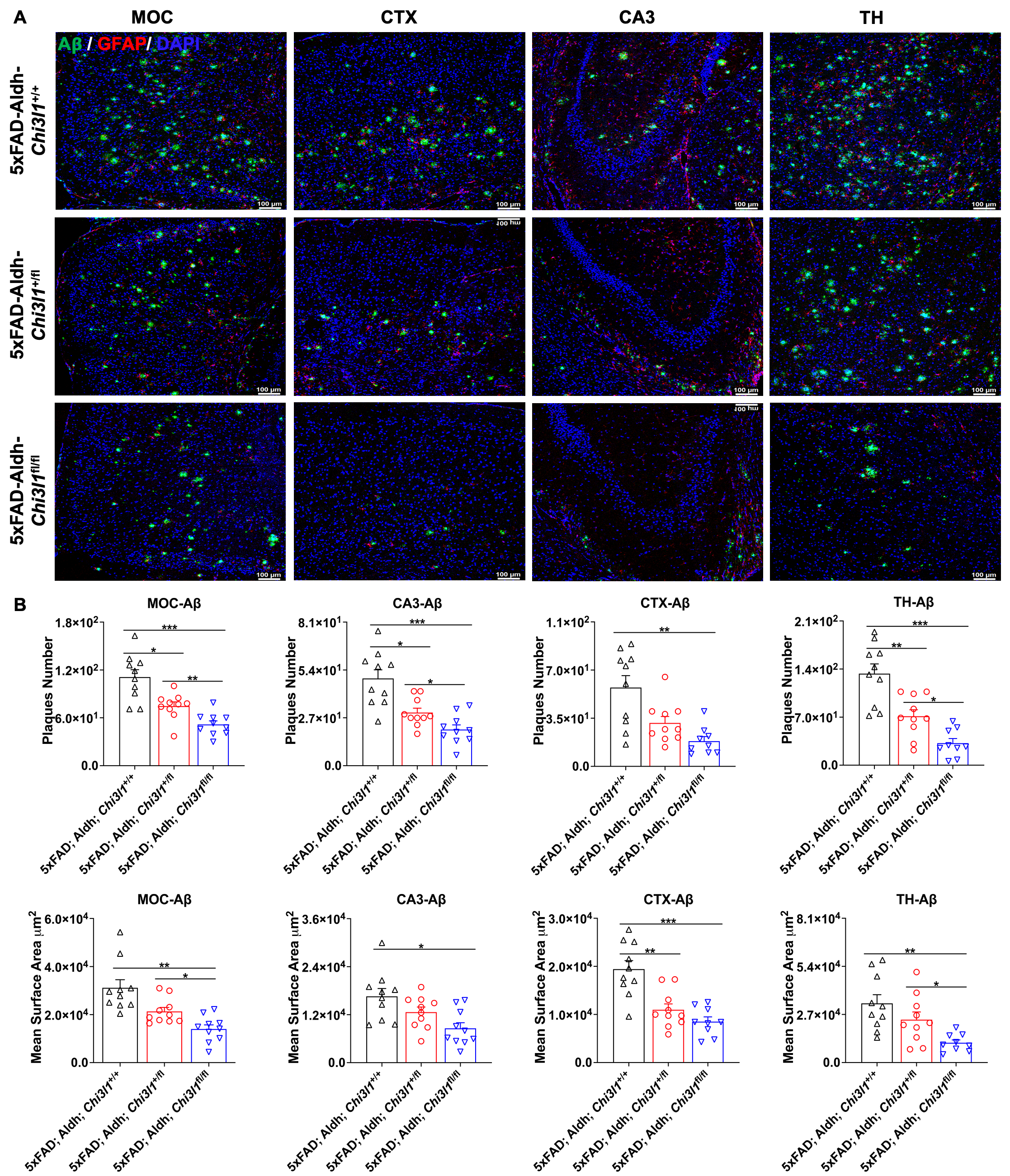


**Fig. S4. Knockout of YKL-40 in astrocytes reduces Aβ deposition in 5xFAD mice brain.** All mice were IP injected with 20 mg/ml tamoxifen in corn oil (100 µl per mice/per day) for 7 consecutive days before experiments. Experiments were carried out 7 days after last tamoxifen injection. **A.** Confocal images (Mag. 10X) of motor cortex + metrosplenial cortex (MOC), mortex (CTX), Cornu Ammonis 3 (CA3), and thalamus (TH) regions of 5xFAD;Aldh;*Chi3l1*^+/+^; 5xFAD;Aldh;*Chi3l1*^+/fl^; 5xFAD;Aldh;*Chi3l1*^fl/fl^ mouse brain immuno-stained with DAPI (blue signal), anti-Aβ (green signal), and anti-GFAP (red signal) antibodies. Scale bars, 100 μm. **B.** Quantification of plaque number from Aβ signals and mean surface area. Data are mean ± SEM. *One-way ANOVA* with *Tukey’s post hoc* comparisons. **p* < 0.05, ***p* < 0.01, ****p* < 0.001 compared to 5xFAD;Aldh;*Chi3l1*^+/+^.

**C**

**
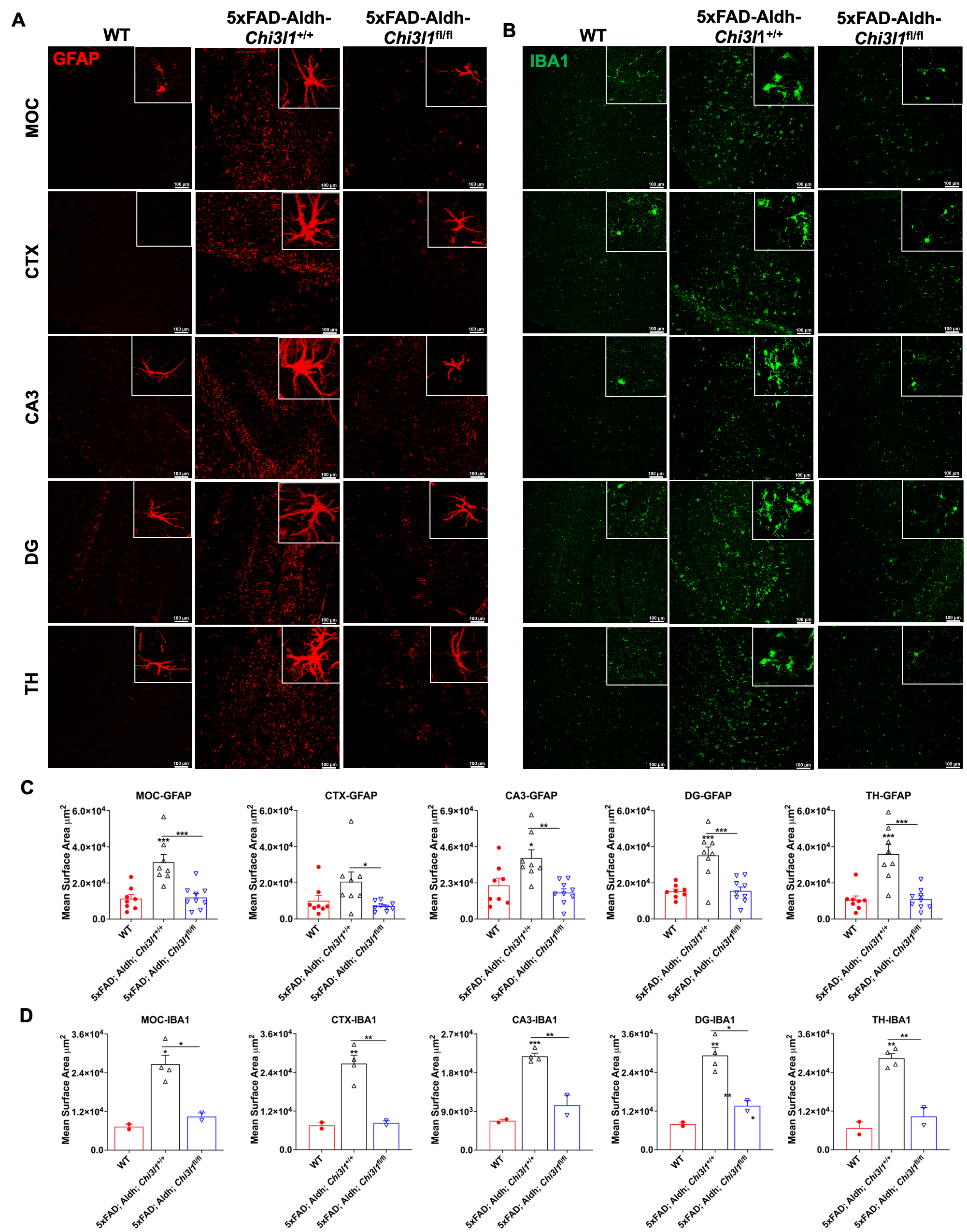
**

**
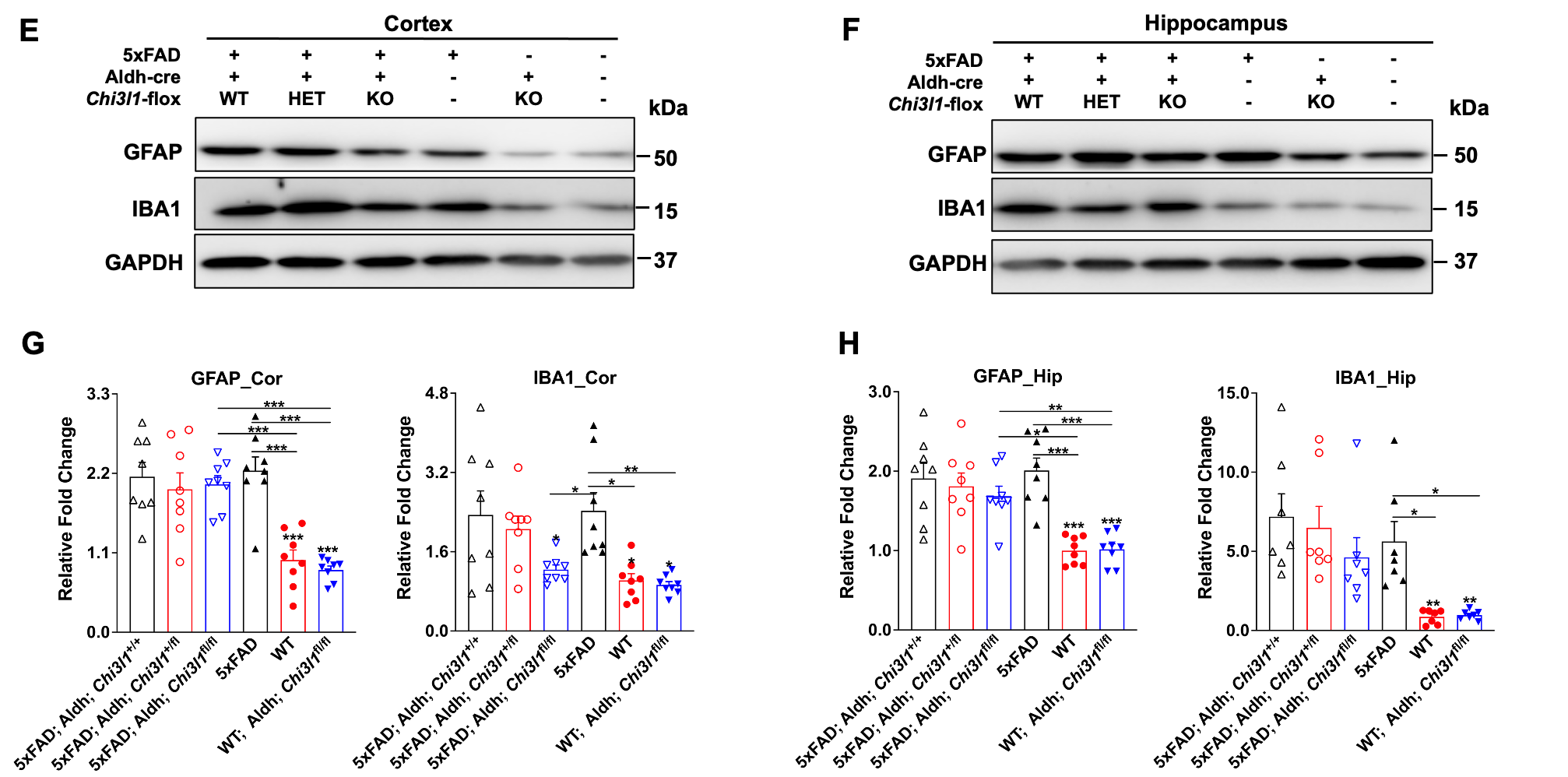
**

**Fig. S5. YKL-40 depletion in astrocytes reduces glial activation.** All mice were IP injected with 20 mg/ml tamoxifen in corn oil (100 μl per mice/per day) for 7 consecutive days before experiments. Experiments were conducted 7 days after last tamoxifen injection**. A-B.** Confocal images (Mag. 10X) of 7m 5xFAD mice brain immuno-stained with DAPI (blue signal), anti-IBA1 (green signal), and anti-GFAP (red signal) antibodies. Scale bar, 100 μm. **C-D.** Mean surface area was quantified. n=2 to 4. Data are mean ± SEM. **E-F.** Western blotting analysis of GFAP, and IBA1 expression from cortex (Cor) and hippocampal (Hip) lysates. GAPDH was used as an internal control. **G-H.** Quantification results of Western blot analysis are shown. Data are mean ± SEM. *One-way ANOVA* with *Tukey’s post hoc* comparisons. **p* < 0.05, ***p* < 0.01, ****p* < 0.001.


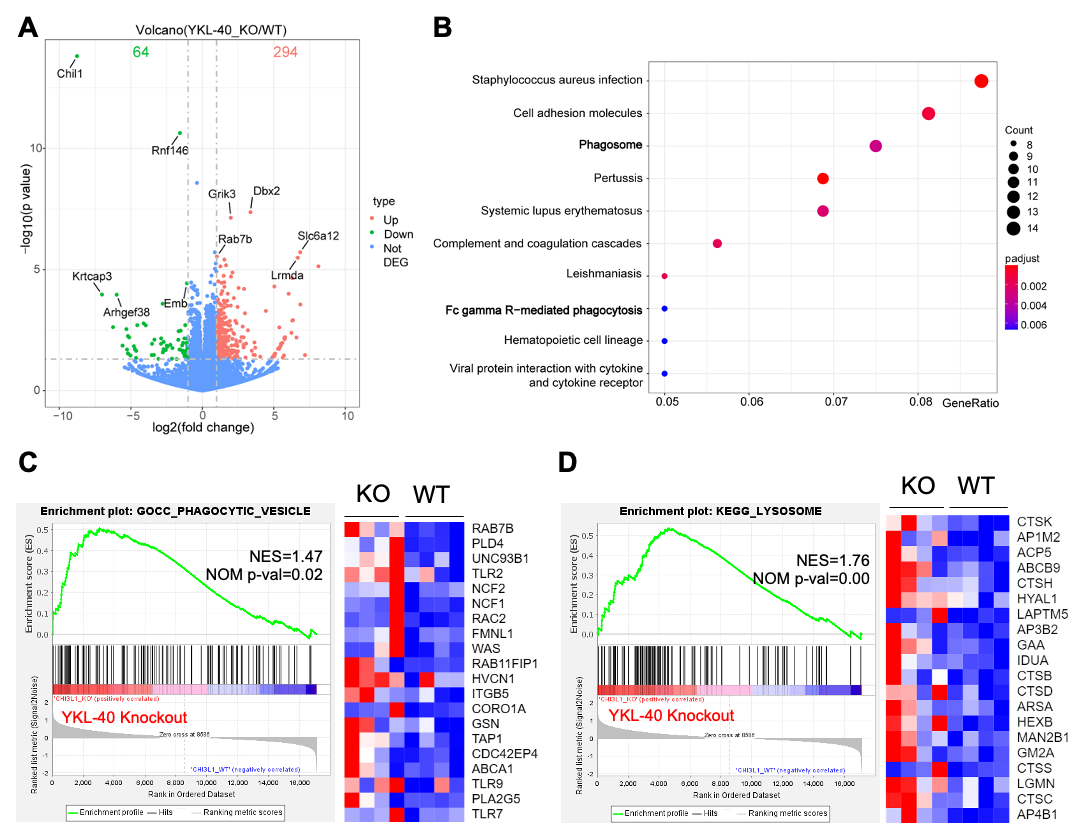


**Fig. S6. Transcriptomic analysis of YKL-40 knockout astrocytes.** **(A)** The volcano plot shows the 258 differentially expressed genes (294 up-regulated and 64 down-regulated, Foldchange>2, p<0.05) between WT and *Chi3l1*^-/-^ (KO) in primary astrocytes by RNA-seq. **(B)** KEGG enrichment analysis of all DEGs showed that KO-associated with phagosome signaling pathway. GSEA analysis showed that genes associated with **(C)** phagocytic vesicles and **(D)** lysosome signaling were significantly up-regulated in KO astrocytes.
